# Adaptive Responses of Tropical Crops: A Multi‐Scale Omics Integrated Perspective

**DOI:** 10.1002/advs.76735

**Published:** 2026-07-27

**Authors:** Peilin Wang, Chenhui Li, Dongjiao Wang, Qibin Wu, Zhen Zeng, Huiming Guo, Han Cheng, Songbi Chen, Hongxing Cao, Hongmei Cheng, Jianghui Xie, Youxiong Que

**Affiliations:** ^1^ State Key Laboratory of Tropical Crop Breeding Institute of Tropical Bioscience and Biotechnology Chinese Academy of Tropical Agricultural Sciences Sanya Hainan China; ^2^ Academician Workstation, National Nanfan Research Institute Chinese Academy of Agricultural Sciences Sanya China; ^3^ National Engineering Research Center for Sugarcane Key Laboratory of Sugarcane Biology and Genetic Breeding Ministry of Agriculture and Rural Affairs, College of Agriculture Fujian Agriculture and Forestry University Fuzhou Fujian China; ^4^ State Key Laboratory of Tropical Crop Breeding Rubber Research Institute Chinese Academy of Tropical Agricultural Science Haikou Hainan China; ^5^ State Key Laboratory of Tropical Crop Breeding Tropical Crops Genetic Resources Institute Chinese Academy of Tropical Agricultural Sciences Haikou Hainan China; ^6^ State Key Laboratory of Tropical Crop Breeding Coconut Research Institute Chinese Academy of Tropical Agricultural Sciences Haikou Hainan China

**Keywords:** agroecological strategies, climate‐resilient, genomic adaptation, stress resilience, tropical agriculture

## Abstract

Tropical regions sustain crops essential to global food security and economic development, notably sugarcane, banana, cassava, rubber and oil palm, yet these species face extreme and variable environments. This review synthesizes their adaptive strategies shaped by long‐term natural selection and domestication. We examine how genomic features underlie crop complexity, how structural variation drives key adaptive traits, and how domestication and breeding reshape genomes to enhance yield while redefining adaptive trajectories. Future efforts should be devoted to resolving synergistic stress‐response mechanisms via multi‐omics, accelerating stress‐resilient cultivar development using wild germplasm and molecular design breeding, optimizing tropical agroforestry through niche‐based frameworks, and applying tropical adaptive mechanisms to other crops via synthetic biology and gene editing. Collectively, these advances provide theoretical and practical strategies for advancing climate‐resilient, sustainable tropical agriculture.

## Graphical abstract

Tropical crops integrate genomic, morphological, physiological, and ecological adaptations to thrive under extreme and variable environments. This review highlights how natural selection, domestication, and breeding shape stress resilience, resource‐use strategies, and productivity in sugarcane, banana, cassava, rubber and oil palm, offering new routes toward climate‐resilient and sustainable tropical agriculture.

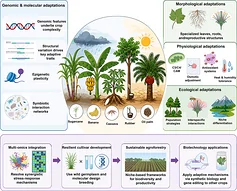

## 1 Introduction

Tropical regions, located between 23.5°N and 23.5°S, are home to some of the Earth's richest biodiversity, supporting human livelihoods and economic development [1]. These regions are key producers of crops like sugarcane (*Saccharum* spp.), rubber (*Hevea brasiliensis*), banana (*Musa acuminata*), cassava (*Manihot esculenta*), cocoa (*Theobroma cacao*), yellow pitaya (*Selenicereus megalanthus*) and oil palm (*Elaeis guineensis*), which are essential in global agri‐food systems. However, tropical agriculture faces unique environmental challenges, including frequent extreme weather events, strong sunlight, high temperatures, seasonal drought, poor soils and severe pest and disease pressure [2]. Importantly, these stresses do not occur independently. Instead, they interact in characteristic ways [3, 4, 5]. For example, high temperatures often coincide with high humidity, strong sunlight is typically followed by drought, and poor soils frequently interact with aluminum toxicity and pathogens. A quintessential example of a multi‐stress unique to tropical systems is the concurrent occurrence of high temperatures, high humidity and waterlogging during monsoon seasons, a scenario rarely faced by temperate crops. Under such conditions, root hypoxia is aggravated by heat‐accelerated respiration and humidity‐favored pathogen proliferation, creating synergistic damage that cannot be predicted from single‐stress responses. Currently, studies on such multi‐stress combinations in tropical crops remain surprisingly limited [3, 4, 5]. Addressing these multi‐stress scenarios is therefore a pressing priority for future research. This combination of stresses is a major obstacle for tropical crops, making them different from temperate crops. It has become a critical scientific challenge to know how these crops survive and thrive in such conditions, and how they connect traditional ecology with modern molecular biology.

Characterized by year‐round warmth (annual mean temperature ≥ 20°C), strong solar radiation and limited diurnal temperature variation, tropical climates create unique constraints on the balance between photosynthesis and respiration [6]. Precipitation varies widely, from humid rainforests to dry savannas, exposing crops to cycles of drought and waterlogging [7]. Intense weathering and leaching make many tropical soils acidic and nutrient‐poor, with iron and aluminum oxides immobilizing phosphorus and further limiting nutrient availability [8]. Hot and humid conditions also lead to frequent pest outbreaks, while coastal typhoons cause damage and accelerate soil erosion. Intense interspecific competition often promotes allelopathic interactions [9].

During the development of global agriculture and economic development, tropical crops have diversified into numerous lineages, mainly through natural selection and domestication. Sugarcane, a key crop for sugar and bioenergy, exemplifies how tropical adaptation can transform regional economies and global markets [10]. In addition, tropical agriculture includes high‐value crops like banana, cassava, cocoa and coffee (*Coffea arabica*) [11, 12, 13], industrial crops like rubber tree and oil palm [14, 15], and staple crops like rice and maize [16, 17]. Especially, high‐value fruit and trade crops, including banana and mango (*Mangifera indica*), are crucial for rural livelihoods and global consumer markets [18]. These crops support global food security, industrial raw materials, and international trade, but their production systems also have significant impacts on the environment [19]. Understanding how tropical crops adapt to complex environments is essential for balancing agricultural productivity with ecological sustainability and promoting responsible intensification.

Recently, research on tropical crop adaptation has shifted from eco‐physiological frameworks to genomics‐driven approaches, with the former focus on phenotypic plasticity, the latter uncover the molecular and genetic bases [20]. This has led to the identification of genes and pathways involved in responses to heat, drought, and waterlogging [21, 22], and the development of high‐quality genome assemblies for major crops, laying the groundwork for mechanistic studies and precision breeding [23, 24]. Nevertheless, several critical gaps remain. First, the regulatory networks that help crops withstand combined stresses like heat and drought are still not fully understood [4]. Second, the genetic bases of adaptive traits shaped by evolution and domestication have not been thoroughly explored, and elite alleles in wild relatives are underutilized [25]. Finally, predicting how adaptive mechanisms will perform under future climates and strengthening crop resilience through methods like molecular design breeding are a major challenge for sustainable tropical agriculture [26]. This review aims to synthesize the adaptive mechanisms of tropical crops in response to stress, integrating evidence from morphology, physiology and molecular genetics and to build a connected framework that provides theoretical and strategic directions for addressing climate change challenges. It will also explore how these adaptations can be transferred to other genetic backgrounds, thereby extending the survival strategies of tropical crops to broader agricultural systems.

## 2 Genomics Research on Tropical Crops

**FIGURE 1:**
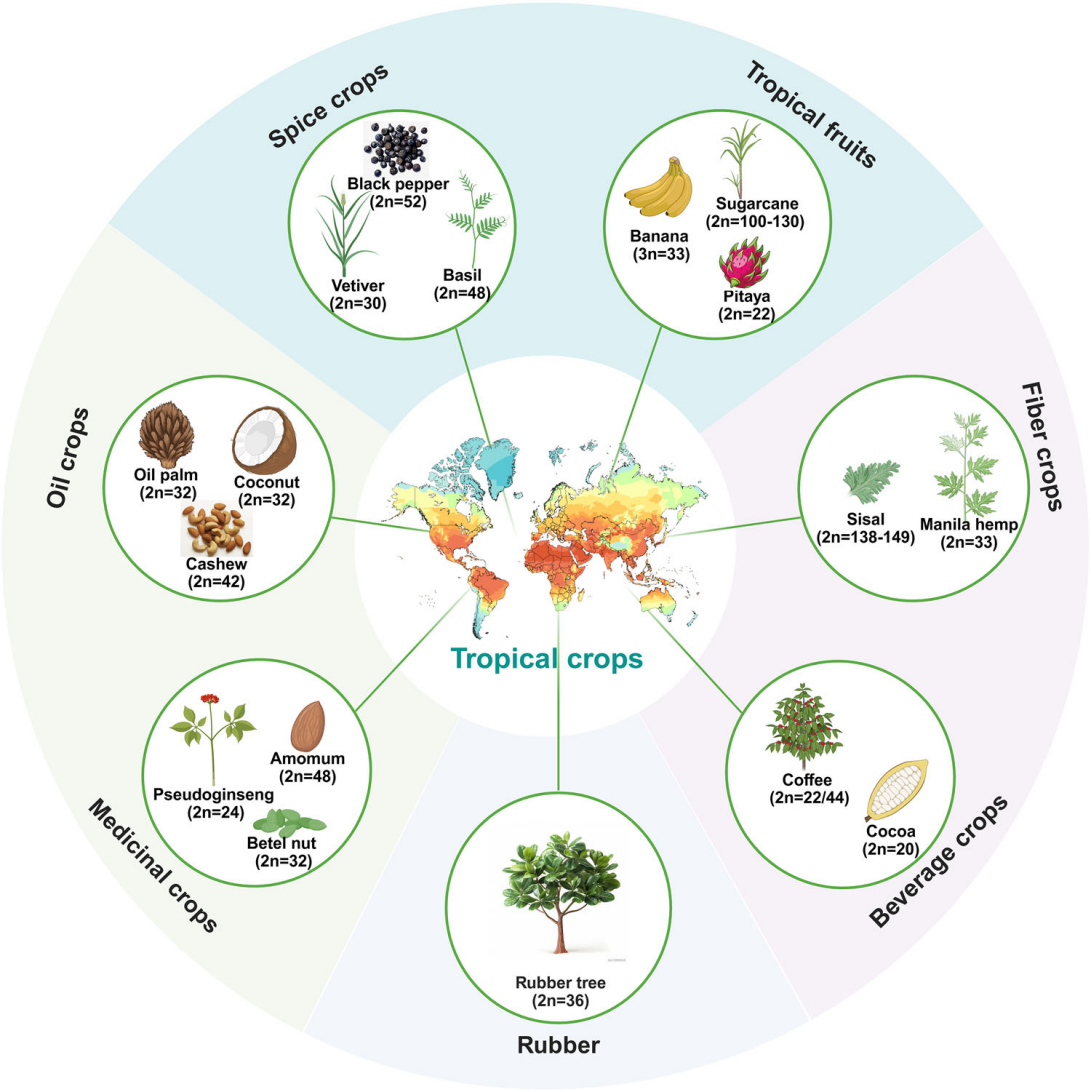
Basic classification of tropical plants and their corresponding chromosomal characteristics. Global distribution and major categories of tropical crops with representative species and their chromosome numbers. Tropical crops are classified into seven major groups, including spice crops, tropical fruits, fiber crops, beverage crops, rubber, medicinal crops and oil crops. Representative species within each group are illustrated, together with their somatic chromosome numbers (2n). Figure created with BioRender.com.

Tropical crops are extraordinarily diverse, not only in their uses but also in the complexity and variation of their genomes. Chromosome numbers range from 20 in cocoa to about 100–130 in sugarcane and 138–149 in sisal (Figure 1). This genomic diversity is closely tied to long‐term adaptation to tropical environments, which are characterized by high temperatures, strong sunlight, abundant rainfall, nutrient‐poor soils and high pest and disease pressure. Processes like polyploidization, expansion of repetitive sequences and chromosomal rearrangements generate redundant gene copies and novel regulatory structures, enhancing physiological flexibility and genetic buffering under complex stresses. Representative tropical crops, including banana, cassava, sugarcane, rubber tree and oil palm, have been extensively characterized at the genomic level, and their key agronomic traits, such as heat tolerance, high photosynthetic efficiency, and disease resistance, along with experimentally validated functional genes, are summarized in Table 1.

**TABLE 1:** Representative genomic advances and adaptive genetic targets in major tropical crops.

Crop	Recent genomic advances	Key agronomic traits	Representative validated functional genes	Biological function
Banana	Reference and wild ancestor genome assemblies [28, 29]; triploid and haplotype‐resolved genomes revealing subgenome evolution, structural variation, and disease‐resistance loci [64, 65]; GWAS and multi‐omics resources for Fusarium wilt resistance and fruit quality [71, 73, 74, 75, 76, 77]	Heat tolerance; high biomass production; Fusarium wilt TR4 and Black Sigatoka resistance; fruit quality and postharvest performance	*MaHSFA2c, MaDREB1F, MaRGA2, MaNAC1, MaACS1*	Heat response; photoprotection; Fusarium wilt resistance; drought tolerance; ethylene biosynthesis and fruit ripening
Cassava	Reference genome and wild/cultivated cassava genome resources [33, 34, 35]; population resequencing and heterozygosity variation analysis [322]; pan‐genome/SV resources supporting adaptive allele discovery [25, 43, 57, 58]	Heat tolerance; drought tolerance; high photosynthetic efficiency; starch accumulation; CMD and CBSD resistance	*MeHsfA2a, MeSWEET10a, MeNBS‐LRR, MeAGPS1a, CYP79D1/CYP79D2*	Heat adaptation; carbon metabolism; drought response; disease resistance; starch biosynthesis; cyanogenic glucoside metabolism
Sugarcane	Monoploid reference genome and polyploid genome resources [23, 24, 27, 309]; super‐pangenome graph, dosage‐aware trait mapping, haplotype‐resolved genome, and breeder‐favored haplotype identification [59, 60]	Heat tolerance; drought tolerance; waterlogging tolerance; high C4 photosynthetic capacity; sucrose accumulation	*TB1, SUS2, WRKY2, SOS1, VPP1, PIP2*	Abiotic‐stress adaptation; sucrose accumulation; sugar metabolism; drought response; water transport
Rubber tree	Chromosome‐level genome assembly and comparative genomic resources; transcriptomic/epigenomic resources for latex production, stress adaptation, and disease resistance [15, 261, 323, 324]	Heat tolerance; drought tolerance; latex yield; disease resistance	*HbWRKY27, HbNAC1, HbERF‐IXc5, HbHMGR1, HbSRPP*	Stress signaling; drought adaptation; ethylene‐mediated latex production; rubber biosynthesis; latex particle stability
Oil palm	Reference genome assembly and resequencing resources [51]; GWAS/QTL mapping for oil yield and Ganoderma resistance [323, 325, 326]; integrated transcriptomic stress‐response networks [66]	Heat tolerance; waterlogging tolerance; nutrient‐use efficiency; basal stem rot resistance; oil yield	*EgHSFA3, EgDREB1, EgPHT1, EgNAC6, EgWRKY40*	Heat response; drought adaptation; phosphorus uptake; stress regulation; disease defense

### 2.1 Genomic Complexity

The genomes of tropical crops vary widely in size, showcasing remarkable structural diversity. For example, sugarcane has an exceptionally large and complex genome, estimated at around 10 Gb [23, 27]. In contrast, crops like banana have moderately sized genomes (∼500–600 Mb) [28, 29], while cocoa (∼430 Mb) and papaya (∼372 Mb) have more compact genomes [30, 31]. These variations are not simply due to gene number but are primarily influenced by polyploidization histories and the buildup of repetitive sequences.

Modern cultivated sugarcane originated from the hybridization of *S. officinarum* (2n = 80) and *S. spontaneum* (2n = 40–128), resulting in an allopolyploid genome with ∼100–130 chromosomes [23, 24]. Polyploidization increases gene copy number, creating redundancy. Some duplicates retain ancestral functions, while others undergo neo‐ or subfunctionalization, expanding adaptive traits [32]. Cassava has a haploid genome of ∼750 Mb and is diploid (2n = 36), characterized by high heterozygosity and many repetitive elements [33, 34, 35]. In banana, triploid cultivars (e.g., AAA and AAB) are common. Triploidy confers sterility, but clonal propagation preserves favorable hybrid combinations and traits like seedlessness and increased biomass [36].

Pitaya represents an emerging tropical fruit crop with unique genomic features that reflect its adaptation to arid and semi‐arid environments. The diploid red pitaya (2n = 2x = 22) has a genome size of approximately 1.41 Gb, which is substantially larger than that of cocoa or papaya, primarily due to the expansion of repetitive elements [37]. Recently, a telomere‐to‐telomere (T2T) gap‐free genome of pitaya was reported, together with an efficient transcript purification system that overcomes viral contamination issues in transcriptomic analyses [38]. More recently, a high‐quality chromosome‐level genome assembly of the autotetraploid yellow pitaya (2n = 4x = 44), harboring 27,246 high‐confidence genes derived from diploid ancestors [39]. These polyploidization events and transposable element expansions have likely contributed to pitaya's remarkable adaptability to heat and drought stress, including its characteristic stem succulence and crassulacean acid metabolism (CAM) photosynthesis.

Transposable elements (TEs) are key drivers of adaptive evolution [40]. Repeats, especially LTR retrotransposons (LTR‐RTs), contribute to genome expansion and generate structural variation (SV) and regulatory innovation. For example, over 90% of the sugarcane genome consists of repeats [27], while cocoa, despite its compact genome, contains around 25% repeats [30]. Stressors like drought or heat can activate transposons, with insertions potentially altering gene expression or even regulatory relationships, providing a source of rapid genetic variation for selection [41, 42]. As recently highlighted in the context of super‐pangenomics and stress breeding, TE activation represents a bidirectional process in which stress induces genomic instability and the resulting insertions simultaneously generate adaptive variation [43].

Adaptive evolution is evident in the expansion of key gene families and regulatory changes. In sugarcane, stress‐responsive genes like heat shock proteins, antioxidant enzymes and late embryogenesis abundant (LEA) proteins show broader expression and greater diversity in more tolerant cultivars [44, 45, 46]. In banana, the expansion of multiple transcription factor (TF) families reflects the complexity of fruit development, ripening control and environmental responsiveness [28]. Banana ripening, which is ethylene‐dependent, benefits from whole‐genome duplications that promote the expansion and retention of genes involved in ethylene biosynthesis and signaling, with stage‐specific expression during ripening [47]. In cassava, the expansion of cytochrome P450 (CYP) genes in the cyanogenic glucoside (CG) biosynthetic pathway supports anti‐herbivore defense [48]. Crops like cocoa and coffee accumulate secondary metabolites such as theobromine, caffeine and flavonoids. Enzyme genes involved in these pathways, often organized in clusters, have diversified via duplication and divergence into multigene families, enhancing metabolic complexity and efficiency [30, 49].

### 2.2 Linking Genomic Complexity to Adaptive Traits

Genomic structural complexity is vital to phenotypic adaptation by influencing the ability to perceive, integrate, and respond to environmental signals, as well as maintain metabolic homeostasis and make rapid regulatory adjustments under variable stress conditions. In tropical crops, TF families such as APETALA2/ethylene‐responsive factor (AP2/ERF), basic leucine zipper (bZIP), NAM/ATAF/CUC (NAC) and WRKY motif‐containing (WRKY) are often expanded. Stress‐responsive families like AP2/ERF and bZIP act as central hubs, converting environmental cues into transcriptional reprogramming [50]. In oil palm, NAC TFs, linked to development and stress responses, are particularly abundant, providing a regulatory foundation for environmental adaptation [51].

Beyond TFs, receptor‐like kinases (RLKs), receptor‐like cytoplasmic kinases (RLCKs) and mitogen‐activated protein kinase (MAPK) cascades form the core “perception–amplification–transcriptional reprogramming” signaling axis. In highly polyploid crops, these gene families tend to expand and subfunctionalize, enhancing responses to fluctuations in soil moisture, nutrient availability and pathogen pressure [32, 52]. For example, leucine‐rich repeat receptor‐like kinases (LRR‐RLKs) in sugarcane show significant duplication and diversification associated with development, circadian stability and disease defense [53, 54, 55].

The sources of genomic complexity vary among tropical crops. In cassava, complexity arises from a variability‐dominated architecture shaped by high heterozygosity, abundant repeats, and extensive SV [34, 56]. Among cassava populations, there are widespread single‐nucleotide polymorphisms (SNPs) and high heterozygosity, along with variable genome features like presence/absence variation (PAV) that emerge during domestication and geographic differentiation. Additionally, LTR‐RT insertions can interact with local epigenetic changes, modulating tissue‐specific or stress‐induced expression of nearby genes [25]. Capturing such PAV and structural variation across tropical crop species requires pan‐genome or super‐pangenome approaches that integrate multiple genomes rather than relying on single references [43, 57, 58]. For sugarcane, a recent multiscale super‐pangenome graph integrating nine chromosome‑level genomes from different ploidy levels and species has captured over 80% of the genomic diversity and enabled dosage‑aware mapping of agronomic traits [59]. More recently, a fully phased genome assembly of the foundational cultivar POJ2878 resolved 118 chromosomes and, together with identity‐by‐descent and allele‐specific expression analyses, identified breeder‐favored haplotypes, including a SUS2 haplotype associated with enhanced sucrose content [60].

Gene family expansion, duplication, and polyploidization provide redundancy and fault tolerance to metabolic networks. When one gene copy is affected by mutation or environmental stress, others can compensate, maintaining the stability of essential pathways [32]. This buffering capacity is crucial for high‐yield tropical crops. In sugarcane, for instance, multiple copies and alleles of key C4 photosynthetic enzymes help maintain high carbon fixation efficiency under intense light and high temperatures [61, 62, 63], strengthening genetic robustness in unpredictable environments.

Epigenetic mechanisms add a rapid, reversible layer to complex genomes. DNA methylation, histone modifications and non‐coding RNAs can reprogram gene expression without changing DNA sequence, offering a key route for short‐term environmental adaptation [32]. In both sugarcane and banana, changes in epigenetic marks and small‐RNA networks can quickly adjust stress‐responsive gene expression under salt and drought stress. Stress‐induced transposon activation, through insertions that modify nearby gene expression or rewire regulatory networks, also generates genetic and epigenetic variation [41, 42]. Structural variation and repeat insertions influence regulation at disease‐resistance loci, revealing subgenome‐ and haplotype‐specific differences in gene expression and response [64]. This landmark study, by constructing subgenome‐resolved haplotype maps of triploid cultivated banana, for the first time systematically revealed in a polyploid crop how structural variations and repeat insertions directly cause the loss of resistance gene clusters and regulatory alterations due to transposon insertions in promoter regions. It further demonstrated subgenome‐specific functional divergence between disease resistance and fruit quality. These findings elevate structural variation from “genetic noise” to a core driving force determining resistance phenotypes, and also provide a key theoretical basis for precise disease resistance breeding through the interplay of epigenetic mechanisms and structural variation. Independently, using two haplotype‐resolved genome assemblies of AAB allotriploid bananas, structural variations and repeat insertions at disease‐resistance loci also contribute to subgenome‐ and haplotype‐specific expression and response patterns [65]. In oil palm, multi‐stress transcriptomic analyses have identified a core response network shared across drought, salinity, heat and cold stresses, highlighting the integrated regulatory systems that enable coordinated multi‐stress responses in perennial tropical crops [66].

### 2.3 Domestication and Breeding Shape Genomic Adaptation

Human domestication and modern breeding have significantly shaped tropical crop genomes, leveraging intrinsic features such as outcrossing, hybridization, polyploidy and clonal propagation. Directional selection on specific agronomic traits consolidates domestication‐syndrome phenotypes [36, 67]. Typically, domestication involves strong selection at a few loci and weak, diffuse selection across many loci, with breeding targets evolving in response to changing production systems and market demands [68, 69].

In crops like banana and cocoa, domestication traits such as enlarged fruits, reduced seeds, improved flavor and reduced fruit drop have been mapped to key genes [67]. In banana, selection on genes related to seed development, parthenocarpy, and sterility led to the emergence of triploid seedless cultivars [28, 36]. Domesticated bananas result from hybridization of multiple wild genetic groups, followed by clonal propagation, which is central to their adaptation [29]. In cocoa, strong selection signals have been found in genes related to lipid biosynthesis, flavor metabolism and pod traits, aiding precise breeding [30, 67]. Similarly, papaya domestication highlights stepwise selection on fruit traits and reproductive systems, demonstrating diverse domestication pathways across tropical crops [31].

Most often, domestication involves bottlenecks that reduce diversity in other regions, potentially accumulating deleterious alleles and limiting pest resistance and climate adaptability [68, 69]. In contrast, wild relatives and landraces retain diverse stress‐resilience alleles shaped by natural selection. In sugarcane, breeding has involved continual introgression and recombination, with large‐scale genomic studies revealing underutilized wild diversity that could enhance stress tolerance and yield [23]. Wild germplasm remains crucial for addressing future biotic and abiotic stresses [70]. For example, wild banana (*Musa acuminata* ssp. *malaccensis*) is a key source of resistance to *Fusarium wilt* TR4, with successful introduction of resistance genes into Cavendish cultivars [71]. In the Amazon, wild cocoa populations harbor genes for disease resistance and drought tolerance, vital for future improvements [72]. Nowadays, adaptive alleles can be precisely incorporated into elite cultivars through marker‐assisted selection (MAS), genomic selection (GS) or genome editing. Even in crops with clonal propagation, hybridization, and polyploidy, genome‐wide association studies (GWAS) have proven effective for precision breeding [73, 74]. In tropical crops, recent CRISPR/Cas9 applications illustrate the potential of precision breeding. In cassava, editing of *CYP79D1* and *CYP79D2* eliminated toxic cyanogen production [75]. In banana, *autoinhibited calcium ATPase* (*ACA*) genes, as negative immunity regulators, were candidate editing targets for resistance breeding [76, 77].

## 3 Morphological and Structural Adaptations of Tropical Crops

**FIGURE 2:**
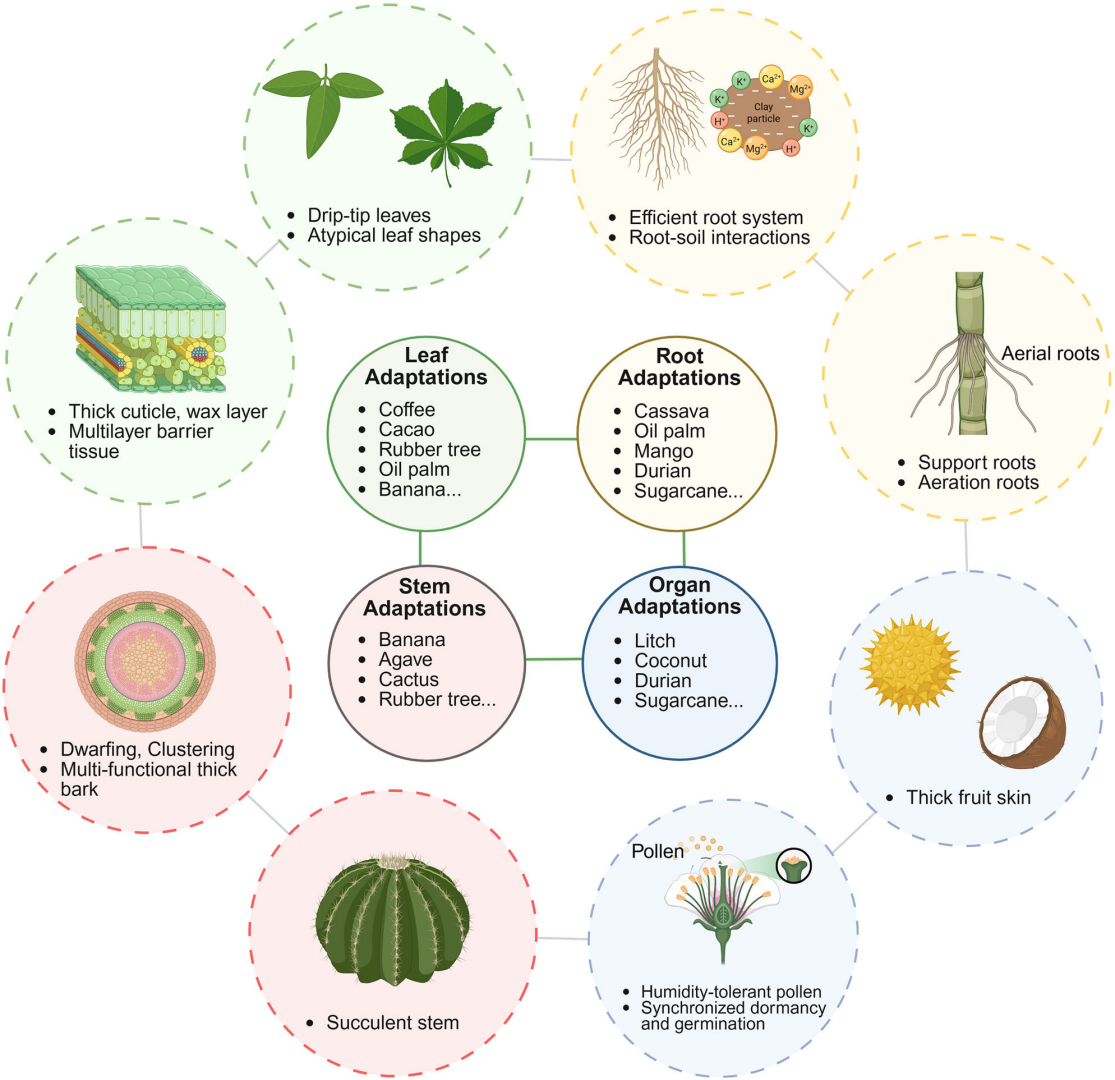
Morphological adaptations of leaves, stems, roots, and reproductive structures in tropical crops. Schematic overview of morphological and organ‐level adaptations in tropical crops. Adaptations are summarized across four major plant components, including leaf adaptations, root adaptations, stem adaptations and reproductive or storage organ adaptations, with representative tropical crop examples listed for each category. Illustrative traits include thick cuticle and wax layers and multilayered barrier tissues, leaf tips bearing water droplets and unusual leaf shapes, shallow fine root networks and root–soil interactions, aerial roots supporting aeration and mechanical stability, dwarfing and clustered growth with thick multifunctional bark, succulent stems, thick fruit skins and pollen and phenology traits associated with humid environments, as well as synchronization of seed dormancy and germination. Figure created with BioRender.com.

As we know, the survival and reproduction of tropical crops reflect long‐term co‐evolution with, and domestication‐driven selection under, stresses such as high temperature, intense radiation, heavy rainfall and waterlogging, nutrient‐poor acidic soils and strong winds (Figure 2). Under these multi‐factorial pressures, morphological and structural traits often constitute the first physical line of defense against environmental challenges and, together with physiological and metabolic regulation, determine overall fitness and yield stability.

### 3.1 Leaf Morphology

Leaves, as the primary organs for energy capture and gas exchange, reflect long‐term adaptations to tropical climates in both morphology and anatomy. Tropical environments impose pressures like high temperatures, intense radiation, and heavy rainfall, shaping leaf size, thickness, angle and surface features as trade‐offs between energy balance and water economy. In humid regions with ample light, large leaves optimize light interception, while smaller leaves provide better heat dissipation during drought or heat waves [78]. Tropical crops, such as banana, have large leaves to support biomass accumulation, while crops like sugarcane can reduce light interception and transpiration under water limitation through leaf rolling and postural adjustments [78, 79, 80].

A thick cuticle and wax layer are common structural adaptations for heat and drought tolerance. These hydrophobic barriers reflect solar radiation and limit water loss [81, 82]. In sugarcane, cuticle and wax respond to drought and salinity stress [83], while in banana, variation in wax load and composition correlates with leaf water retention [84]. Wax composition also changes across developmental stages and is linked to fatty‐acid elongation and wax‐synthesis gene expression [85]. Stomatal traits and epidermal structures, in combination with wax, form an integrated transpiration‐water conservation system, with traits such as wax and adaxial stomatal density mapped as dehydration‐avoidance mechanisms in banana [86].

A well‐developed palisade mesophyll is suitable for high irradiance and photosynthetic demand. In many tropical crops, leaves contain multiple layers of densely packed palisade cells, maximizing light‐harvesting efficiency and limiting excessive light penetration [87]. Thicker leaves and multilayered palisade tissues optimize internal light distribution, enhancing photosynthesis and reducing photo‐inhibition [88]. In tropical forest plants, plasticity to canopy light gradients is demonstrated by coordinated shifts in traits such as leaf thickness, leaf mass per area (LMA), palisade development and stomatal characteristics [89].

Drip tips are important for managing heavy rainfall and humidity. They reduce water residence time on leaf surfaces by altering tip curvature and drainage pathways, thus stabilizing the leaf‐surface micro‐environment [90, 91]. They can also exude excess internal water and dissolved solutes, maintaining a drier surface [92]. Hydathodes, found in crops like Musaceae and aroid taro (*Colocasia esculenta*), release xylem sap when root pressure is high, functioning as pressure‐relief valves and routes for ion and solute export [93].

Heterophylly, or leaf‐form differentiation. Juvenile leaves are larger and thinner for shaded conditions, while mature leaves exposed to higher light are smaller, thicker and have more developed cuticles and palisade tissues for light tolerance [94]. In response to environmental variation, this plasticity allows crops to efficiently exploit light resources in complex tropical ecosystems with heterogeneous irradiance [89]. The capacity to adjust leaf morphology, characterized by complex canopy structures and highly heterogeneous irradiance, can even enable crops to exploit light resources efficiently within tropical ecosystems.

### 3.2 Root‐System Traits

Combined with leaching, acidic and weathered soils, the strong seasonality of tropical rainfall leads to vertical stratification of soil resources. Surface layers are enriched in nutrients from litter inputs, organic matter and rapid mineralization, while deeper layers serve as water reservoirs [95, 96]. In tropical crops, root systems balance “topsoil nutrient capture” and “deep‐water acquisition” aided by mechanisms like solubilization, detoxification, and microbial facilitation to enhance resource efficiency [97].

Many tropical crops, like sugarcane, develop shallow, lateral root systems to forage for topsoil nutrients in nutrient‐poor, leached lateritic soils [98]. Cassava genotypes adapted to low‐phosphorus conditions show increased shallow‐root length density and greater rhizosphere secretion of organic acids, improving phosphorus (P) acquisition [99, 100]. Phosphate mineral forms affect root exudation and rhizosphere P activation, highlighting root exudation as a key adaptive mechanism in P‐limited soils [101]. In sugarcane, similar strategies involve shallow root distributions to enhance P uptake under low‐P conditions, but this comes with the cost of limited access to deep water during droughts [102]. In contrast, drought‐tolerant coffee develops deeper roots and stronger stomatal regulation to maintain water status during soil drying [103].

Tropical crops can tolerate hypoxic stress from heavy rainfall and waterlogging. In woody plants, aerial and buttress roots address these challenges by altering drainage and enhancing oxygen uptake [104]. In sugarcane, aerenchyma formation and cell‐wall remodeling help with waterlogging tolerance [105]. Similarly, banana forms adventitious roots and induces aerenchyma under waterlogging, restoring oxygen diffusion and activating antioxidant defenses [106]. However, aerenchyma formation, representing a tradeoff between stress tolerance and structural integrity, incurs costs including reduced mechanical strength of root tissues and potentially increased susceptibility to soilborne pathogens [107]. Flooding can trigger aerial roots higher on stems that absorb atmospheric nitrogen and form stem nodules, contributing more fixed nitrogen than roots [108, 109]. Oil palm also develops aeration‐related structures under waterlogging, with variations in aerial root numbers helping mitigate growth suppression [110]. During extreme events like typhoons, buttress roots enhance anchorage and wind stability, whereas reduce uprooting risk in large tropical trees [111, 112]. Similarly, aboveground brace roots (a type of adventitious root) contribute to stalk anchorage and lodging resistance in cereal crops such as maize. Multiple brace root phenotypes including root angle, width, and whorl number influence the mechanical contribution to anchorage, with genotypes exhibiting greater brace root soil contact showing reduced root lodging susceptibility [113, 114, 115]. In sugarcane, similar strategies may involve brace or nodal root architecture that reinforces stem base stability under wind and heavy rain conditions. During extreme events like typhoons, buttress roots, found in large tropical trees, enhance anchorage and wind stability, reducing uprooting risk [111, 112].

Root exudates act as chemical interfaces to confront soil stresses. In acidic tropical soils, symbiosis enhances nutrient acquisition, especially phosphorus. In sugarcane, overexpression of the multidrug and toxic compound extrusion (MATE) transporter *SbMATE* increases citrate and malate efflux, a chelation barrier to reduce aluminum toxicity [116]. Phosphate‐solubilizing bacteria (PSB) and arbuscular mycorrhizal fungi (AMF) improve phosphorus availability and promote growth in tropical crops like sugarcane [117]. In banana, root exudates enhance rhizosphere colonization by plant‐growth‐promoting rhizobacteria (PGPR), strengthening biological control against pathogens [118, 119]. Similarly, cassava relies on AMF for phosphorus acquisition in P‐deficient soils, with mycorrhizal inoculation boosting growth [120, 121]. Root exudates also suppress soil pathogens and reshape microbial communities, promoting biotic resistance and nutrient mobilization [122]. These chemical and symbiotic pathways, together with architectural strategies, form an integrated root‐adaptation framework that supports resource acquisition and yield stability under compound stresses like low P–drought–waterlogging [97].

### 3.3 Stems and Whole‐Plant Architecture

In tropical crops, stems exhibit sophisticated adaptations to physical stresses such as strong winds, heavy rainfall, drought and high temperatures (Figure 2). They mainly involve plant architecture, supporting tissues, and water storage systems, which balance “resistance to lodging and breakage” with “maintenance of growth and yield” [123].

Dwarfing and clumping growth habits are key morphological strategies for coping with strong winds [124]. Due to a reduced center of gravity and optimized stem architecture, dwarf coconut (*Cocos nucifera*) cultivars have greater stability and lower lodging risk under high winds [125]. Molecular breeding studies associate dwarfism with natural variation in gibberellin metabolism genes [126]. In banana, reduced plant height, thicker pseudostems, appropriate stand density and field support management help reduce wind‐induced breakage and lodging [127]. Under production conditions, semi‐dwarf genotypes, differing from wild types in hormone‐ and metabolism‐related pathways, show enhanced tolerance to wind and rain damage [128]. Given the frequency of extreme weather in the tropics, bananas rank among the crops with the highest agricultural losses due to typhoons, making lodging resistance a central breeding objective in major production regions [129].

As reported, where strong winds and heavy rainfall co‐occur, lodging typically manifests as stem breakage or stalk lodging. Accordingly, stem adaptation focuses on enhancing bending stiffness, compressive resistance and basal stability in tropical monsoon systems. In sugarcane, structural defects underlie susceptibility to lodging [130]. Stem mechanical performance is tightly linked to cell‐wall composition, with lignin, cellulose and hemicellulose forming the primary material determinants of lodging resistance [113, 131]. In banana, pseudostem load‐bearing capacity results from leaf‐sheath architecture, fiber bundles surrounding vascular tissues and tissue lignification. Associations among pseudostem diameter, lignin content, and mechanical strength provide a structural basis for improving lodging resistance through cell‐wall–targeted pathways [132]. Aligning with high‐density tropical planting systems, selecting for “compact architecture + thicker pseudostems + tougher tissues” reduces wind‐induced breakage and lodging risk [127].

Succulent stems are a key adaptation to seasonal drought. In tropical savannas and semi‐arid regions, prolonged dry seasons challenge crop survival. Succulence is a specialized drought‐adaptation strategy involving structural and functional remodeling of parenchyma cells [133]. In Agave (*Agave americana* L.), enlarged succulent storage organs store carbon in stems and/or leaf bases and support vegetative propagation [134, 135, 136]. Succulence, frequently linked with CAM, is often paired with compatible solutes to stabilize cellular osmotic potential [137]. CAM crops like Agave, pineapple (*Ananas comosus*), vanilla (*Vanilla* spp.) and cactus (*Opuntia* spp.) integrate structural water storage and metabolic water saving for greater drought resilience [138]. At the molecular level, circadian regulation across transcripts, proteins, and metabolites controls CAM systems, sustaining metabolism while conserving water during dry seasons [139]. Cactaceae crops, such as prickly pear (*Opuntia ficus‐indica*), also rely on succulent stems for water storage, supplemented by specialized mucilage cells rich in polysaccharides, which reduce water loss [140, 141]. Therefore, succulent stems are integrated systems combining water uptake, osmotic storage, metabolic conservation and physical barriers, ensuring survival and productivity during seasonal drought.

### 3.4 Reproductive Organs

Reproductive success is crucial for fitness in tropical crops, yet reproductive organs are highly vulnerable to high temperatures, humidity, rainfall and pathogen pressure. Pollen tolerance to heat and humidity is essential for overcoming reproductive constraints in tropical environments (Figure 2). High humidity can induce pollen water uptake, clumping, premature germination or rupture, while high temperature reduces pollen viability and shortens the effective pollination window [142, 143].

Pollen thermal tolerance is narrower than that of vegetative tissues. Many tropical crops have evolved specialized pollen‐wall architectures and lipid layers that mitigate high‐humidity stress by limiting water retention, buffering osmotic shock and sustaining pollination success [144]. In tropical cash crops, coconut genotypes vary in pollen morphology, exine ornamentation, and viability [145, 146]. In tropical fruit trees, such as lychee (*Litchi chinensis*), pollen form and viability are linked to pollination biology, with genotypes showing differences in pollen yield, viability and germination rates [147]. High humidity suppresses pollen dispersal and can damage exine and coat structures, making cultivar‐level variation in pollen viability ecologically and agronomically important [148].

In addition to intrinsic pollen tolerance, tropical crops buffer environmental uncertainty through floral architecture and pollination ecology. Cocoa flowers, with specialized spatial arrangements of stamens and pistils, rely on specific pollinators, sustaining pollination under wet conditions [149]. Similarly, oil palm, depending on pollinators like weevils, is influenced by rainfall and humidity, which affect pollinator behavior and fruit set [150]. Pollen humidity tolerance in tropical crops is thus an integrated adaptive trait, involving a thickened exine barrier, a hydrophobic lipid coating and regulated genetic networks.

The thick pericarp and/or cuticle, a composite interface for mechanical defense, water regulation, and chemical protection, is a major adaptive strategy [151, 152]. In coconut, the thick exocarp and secreted flavonoids help resist pathogen invasion under high humidity and frequent rainfall [153]. Many tropical fruit peels are rich in terpenoids and phenolics, which have antimicrobial and antioxidant properties [154, 155]. For example, mango peel extracts suppress postharvest pathogens, with abundance correlating to longer shelf life [156, 157].

Seed dormancy is an adaptive mechanism that helps tropical crops cope with seasonal drought and ensure persistence. In regions with seasonal precipitation, seeds break dormancy and germinate after receiving specific environmental cues [158, 159, 160]. This strategy involves diverse physiological and structural bases. Many tropical legumes are physically dormant, with palisade cells in the seed coat forming a water‐impermeable barrier [161]. As an essential strategy, seed dormancy, governed by genetic programs that sense and integrate environmental signals, is a complex adaptive trait, but not a passive arrest in response to unstable tropical rainfall.

In conclusion, tropical crops have adapted to diverse environmental challenges through structural modifications in leaves, roots, stems, and reproductive organs. These traits, shaped by natural selection and domestication, provide insights into plant evolution and offer valuable resources for developing stress‐resilient, high‐yield cultivars through biomimetic design and molecular breeding.

## 4 Physiological and Metabolic Adaptations of Tropical Crops

**FIGURE 3:**
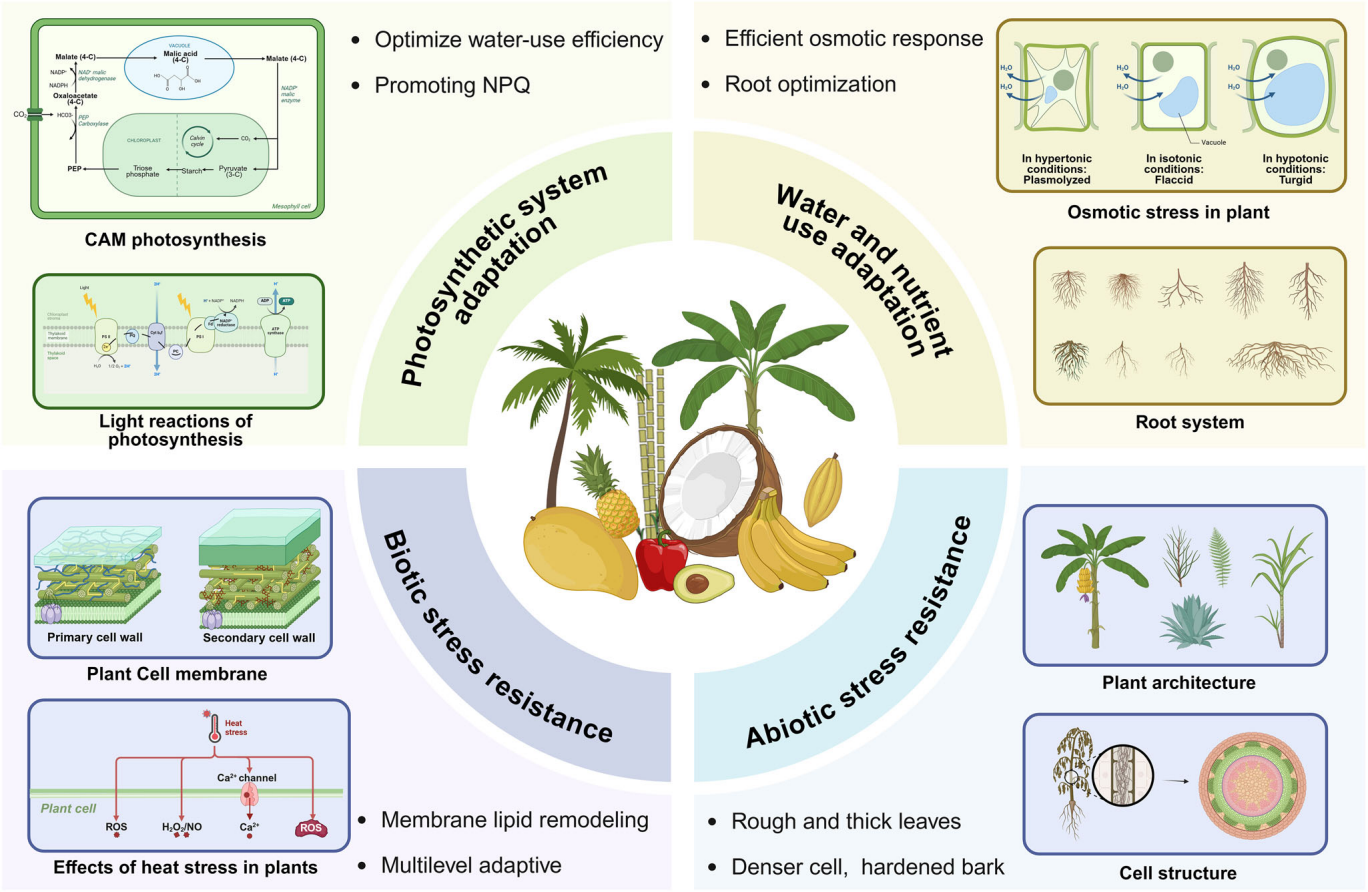
Physiological adaptations of tropical crops. Conceptual summary of major physiological and structural mechanisms underlying stress adaptation in tropical crops. Key adaptive strategies are organized into four modules, including photosynthetic system adaptation, water and nutrient use adaptation, abiotic stress resistance, and biotic stress resistance. Representative processes and traits illustrated include Crassulacean acid metabolism (CAM) photosynthesis and modulation of photosynthetic light reactions to improve water‐use efficiency and protect photosystem II via non‐photochemical quenching (NPQ), osmotic stress responses and root system optimization for complex environments, plant architecture and cellular structural features associated with tolerance, and membrane‐associated adjustments such as lipid remodeling and multi‐level responses to heat and other stresses. Figure created with BioRender.com.

Over evolutionary timescales, tropical crops have developed complex physiological and metabolic strategies that enable growth and reproduction under high temperatures, intense irradiance, water limitation and nutrient‐poor soils. These strategies encompass photosynthesis, water and nutrient use, responses to heat and humidity, and stress‐defense metabolism, forming the core adaptive toolkit for crop performance in tropical environments (Figure 3). Unlike temperate crops, which mainly cope with single‐season stresses, tropical crops have evolved a unique metabolic syndrome to address multiple stresses, such as high temperature and intense light, drought and nutrient‐poor soils and high temperature and high humidity. In this section, we focus on observable physiological and metabolic phenotypes, including photosynthetic performance, osmotic adjustment, antioxidant capacity, membrane stability, water and nutrient use and heat and humidity tolerance.

### 4.1 Photosynthetic Systems

Regarding tropical crops, the emergence of C4 and CAM pathways represents key metabolic strategies to achieve efficient carbon assimilation and water conservation under high temperatures, strong light and periodic water deficits. In C4 crops like sugarcane, CO_2_ concentration is achieved through spatial separation. This reduces photorespiration and enhances carbon gain per unit water and nitrogen investment [162]. Compared with Rubisco, C4‐type Phosphoenolpyruvate carboxylase (PEPC) has a higher affinity for inorganic carbon, sustaining high assimilation rates even under low stomatal conductance [163]. Similarly, stress adaptation in tropical C4 crops is also reflected in the plasticity of decarboxylation routes. Under water limitation, sugarcane increases bypass decarboxylation, maintaining CO_2_‐pump efficiency and assimilation stability [164, 165]. For CAM plants, the temporal separation of carbon fixation especially nighttime stomatal opening and CO_2_ uptake versus daytime CO_2_ reassimilation, inherently reduces transpirational water loss and mitigates heat‐induced oxidative damage. Pineapple, a CAM crop, opens stomata at night, fixes CO_2_ via PEPC and stores it as malate in vacuoles. During the day, stomata close, and malate is decarboxylated to supply CO_2_ to the Calvin cycle, improving water‐use efficiency. Interestingly, CAM evolution in pineapple is driven by regulatory rewiring of pre‐existing metabolic modules and enrichment of cis‐regulatory elements, rather than novel gene acquisition, highlighting regulation‐centered CAM adaptation [166]. Compared to C3 plants, CAM plants fix CO_2_ at night and release it during the day for Calvin‐cycle assimilation, increasing water‐use efficiency by three to six times, forming the core of CAM adaptation to seasonal drought [167, 168, 169, 170]. From above, C4 and CAM represent not only photosynthetic adaptations but also integral components of thermotolerance strategies in tropical crops.

Interestingly, the antioxidant enzyme system serves as the primary physiological barrier against photodamage. Enzymes like superoxide dismutase (SOD), ascorbate peroxidase (APX) and glutathione reductase (GR) work together to form a robust reactive oxygen species (ROS)‐scavenging network. Under high light, SOD activity can increase two‐ to threefold within hours, efficiently neutralizing superoxide radicals generated from over‐reduced electron transport chains [171]. The ascorbate‐glutathione (AsA‐GSH) cycle dynamically adjusts metabolite pools, maintaining or restoring the reduced‐to‐oxidized ratios of AsA and GSH under stress conditions, thereby buffering cellular redox potential and preventing peroxidative damage [172]. Under combined drought and low temperature, drought‐tolerant sugarcane genotypes have higher SOD and APX activities [173]. More generally, SOD, APX, GR and catalase (CAT) activities increase during drought, detoxifying ROS [174]. In banana, drought induces higher H_2_O_2_ and MDA levels, along with increased CAT, APX, GR, peroxidase (POD) and SOD activities [175]. Similarly, drought‐tolerant cassava lines elevate AsA and GSH contents, enhance SOD and CAT activities, and upregulate corresponding genes, reducing membrane lipid peroxidation [176]. The AsA‐GSH cycle detoxifies H_2_O_2_ and also regulates redox signaling and stress responses physiology, maintaining cellular redox homeostasis [172]. In cocoa, photochemical physiological responses to high light and photoinhibition are influenced by functional differentiation between sun and shade leaves [177]. These photoprotective mechanisms engage in extensive physiological cross‐talk: xanthophyll‐cycle intermediates regulate antioxidant gene expression and ROS accumulation modulates non‐photochemical quenching induction, forming a dynamic and integrated adaptive network [178].

High‐light adaptation and heat tolerance represent two tightly connected features of tropical crop adaptation, since intense irradiance and high temperature frequently co‐occur in tropical environments. Representative pathways and candidate genes related to photosynthetic stability, photoprotection, antioxidant defense, osmotic adjustment and heat‐shock responses are summarized in Table 2.

**TABLE 2:** Representative pathways involved in high‐light adaptation and heat tolerance in tropical crops.

Crop group	Major pathway/module	Representative components	Adaptive relevance	References
Banana	HSF/HSP response; flavonoid‐mediated ROS buffering; chlorophyll/ripening regulation under heat	HSFs, HSPs, MaHSF11–MaFLS1/MaF3'5'H1, MaASR3–MaHDT1–MaNIP1, antioxidant enzymes	Enhances thermotolerance, reduces heat/light‐induced oxidative injury, and stabilizes fruit quality under high temperature	[175, 238, 240, 244]
Sugarcane	C4 photosynthesis; heat‐shock response; antioxidant and osmotic‐adjustment pathways	C4NADP‐ME, ABI5, SsHAM3a, EaHSP70, SOD, APX, proline, glycine betaine	Maintains high carbon fixation, photosynthetic stability, membrane protection, and drought–heat resilience under strong irradiance	[162, 163, 164, 165, 173, 185, 186, 233, 237]
Cassava	C3–C4 intermediate photosynthetic features; Hsp70‐mediated heat response; antioxidant and osmotic adjustment	Hsp70 family, MeNADP‐ME3, MeYABBY1, SOD, CAT, APX, AsA, GSH, soluble sugars, proline	Supports high photosynthetic efficiency and buffers combined heat, high‐light and drought stress	[176, 222, 239, 241, 242, 243]
Rubber tree	Stress‐signaling, membrane protection, and latex metabolism‐related stress adaptation	WRKY/NAC/ERF‐type regulators, antioxidant enzymes, membrane‐stability components, latex metabolism‐related proteins	Coordinates perennial growth, stress defense, and latex production under hot and humid tropical environments	[15, 195, 196, 197, 198, 261, 323, 324]
Coffee	Osmotic adjustment; antioxidant metabolism; secondary‐metabolite defense	Proline, glycine betaine, AsA‐GSH cycle, antioxidant enzymes, caffeine‐related metabolism	Protects photosystems and cellular redox balance under drought‐associated heat and high irradiance	[179, 180, 181, 182, 183, 184, 206, 207, 208]
Tropical fruits, including pineapple, pitaya, cocoa, papaya and mango	CAM photosynthesis; photoprotection; antioxidant and phenolic/flavonoid metabolism	PEPC, malate metabolism, xanthophyll cycle, NPQ, carotenoids, AsA‐GSH cycle, phenolics/flavonoids	Improves water‐use efficiency, reduces photoinhibition and oxidative damage, and supports fruit quality under strong light and heat	[37, 38, 39, 166, 167, 168, 169, 170, 177, 178, 216, 217, 218]

### 4.2 Water and Nutrient Use

Under drought stress, many tropical crops perform osmotic adjustment by accumulating compatible solutes, thereby maintaining cellular water balance and protecting metabolic processes. Proline and glycine betaine (GB) are two representative osmoprotectants. In coffee, drought strongly reshapes physiological and metabolic profiles and induces accumulation of osmosis‐related metabolites, such as proline [179]. Typically, proline accumulation is driven by both enhanced biosynthesis and suppressed degradation [180, 181], while glycine betaine is mainly synthesized via the choline oxidation pathway, with its functions extending beyond osmotic adjustment to stabilizing protein structure, preserving membrane integrity and protecting photosystems and cellular redox homeostasis under salinity and drought [182, 183, 184]. In sugarcane, genotypes differ in proline and GB accumulation, as well as in oxidative injury and antioxidant capacity under polyethylene glycol‐simulated drought. These differences correlate with drought‐tolerance phenotypes, supporting osmotic adjustment coupled with antioxidant and membrane protection as a common physiological framework in tropical crops [185, 186].

There is a coordinated xylem anatomy and molecular regulation by optimizing root hydraulic architecture to improve water‐use efficiency. As an example, tropical tree species often show higher fine‐root hydraulic conductivity than temperate species, mostly associated with larger vessel diameters, shorter vessel lengths, and aquaporin regulation [187]. Among aquaporins, plasma membrane intrinsic proteins (PIPs) act as key molecular valves for transcellular water transport [188]. However, high hydraulic conductivity also increases vulnerability to cavitation and embolism [189]. Therefore, tropical crops can balance uptake efficiency and transport safety through embolism repair and/or dynamic aquaporin regulation [190, 191]. In oil palm, drought triggers broad differential expression and network remodeling in transport, hormone signaling, and stress‐response pathways, supporting a model of root‐based stress sensing coupled with transport regulation [192].

Highly weathered tropical soils often trap phosphorus and severely restrict crop productivity. To combat this, cassava actively shifts its root exudation based on phosphate‐mineral solubility. By deploying organic acids and regulating root efflux, cassava dynamically frees trapped rhizosphere phosphorus to fuel nutrient accumulation [101]. Crops also supercharge their internal transport networks to maximize phosphorus uptake and distribution [193]. Fungal partnerships offer another vital lifeline. The fungus *Rhizophagus irregularis* casts extensive hyphal nets to dramatically boost phosphorus capture [194]. Cassava relies heavily on these allies during starvation. Introducing *Rhizophagus clarus* rapidly accelerates growth and yields, while native mycorrhizal communities act as massive nutritional reservoirs. This highlights the urgent need to protect and deploy local fungal networks across phosphorus‐depleted tropical ecosystems [122].

### 4.3 Heat‐ and Humidity‐Tolerance

Tropical crops conquer perpetually hot and humid conditions by orchestrating membrane defense, metabolic shifts, and structural adaptations. Scorching temperatures prompt them to safeguard photosynthetic engines and ignite molecular chaperone networks to manage protein folding. During suffocating waterlogging, they detect oxygen drops to spark anaerobic metabolism launching physical escape strategies to survive.

Scorching heat actively disrupts membrane fluidity and structural organization. To survive, tropical crops aggressively defend membrane stability by adjusting fatty‐acid unsaturation and boosting long‐chain fatty acids to halt leakage and protein breakdown [195]. These physical membrane shifts double as early warning alarms that dictate the strength of subsequent heat‐shock defense networks [196]. Furthermore, sterols and phytosterols fortify membrane compactness and actively steer stress signaling [197]. Since severe heat drastically spikes lipid peroxidation threats, crops deploy robust antioxidant arsenals like AsA and tocopherols to conquer reactive oxygen species and prevent devastating secondary damage [198].

Physiologically, cassava throttles midday water loss by shifting stomatal rhythms. Simultaneously, it stockpiles soluble sugars and proline while weaponizing antioxidant enzymes like SOD and APX to crush oxidative damage [176]. During vulnerable reproductive stages, blistering heat strips coconuts of female flowers and ruins fruit set. Surprisingly, high humidity alters this threshold by shielding pollen‐tube growth from heat damage, effectively rescuing reproductive success [199].

When suffocating waterlogging triggers hypoxic stress, tropical crops like rice activate anaerobic metabolism through precise oxygen sensing and structural shifts to survive [200]. Simultaneously, plants weaponize antioxidant systems and hormone networks involving ethylene and gibberellins to manage oxidative damage and build internal air channels. Deepwater rice evolved a dramatic escape strategy in which submergence causes ethylene to surge, triggering ethylene‐responsive factor‐linked regulation. This process engages gibberellin‐driven cell elongation to rapidly stretch internodes, allowing shoots to break through the water surface and restore aerobic respiration [201]. Collectively, these coordinated responses form the core adaptive framework that enables tropical crops to withstand extreme heat, humidity and seasonal flooding.

### 4.4 Stress‐Resistance Metabolism

Tropical crops use stress resistance metabolism to balance biotic defense and abiotic stress protection. At the physiological level, this involves changes in secondary metabolites, antioxidant capacity, cell wall related compounds and redox buffering. These metabolic phenotypes help crops limit pathogen spread, deter herbivores, reduce oxidative injury and maintain growth under compound stresses.

On the biotic front, sugarcane smut triggers rapid early metabolic reprogramming. Plants reallocate energy and amino acids before heavily investing in cell‐wall reinforcement and lignin production to halt disease progression [202]. Across various pathosystems, pathogen‐induced reactive oxygen species dynamically interact with salicylic acid, jasmonic acid and ethylene signaling [203]. Compared to susceptible ones, banana strains resistant to *Fusarium wilt* exhibit markedly divergent physiological metabolic trajectories. They actively redirect resources toward flavonoids, cell‐wall fortification and antioxidant physiological defenses, while mobilizing distinct signaling pathways [204, 205].

Most often, tropical crops rely on signature secondary metabolites for chemical defense. Coffee accumulates purine alkaloids like caffeine to act as toxic antifeedants while promoting disease resistance and allelopathy [206, 207]. These caffeine and pest interactions flow in both directions. The coffee berry borer successfully detoxifies caffeine using its gut microbiota, proving that plant defenses exert intense selection pressure to drive symbiont‐guided pest adaptation [208]. Furthermore, phenolics and polyphenols surge during pathogen attacks to directly inhibit microbes, deter feeding and supply lignin precursors for cell wall reinforcement. Cocoa polyphenol levels strongly predict resistance to the tea mosquito bug [209]. To combat vascular diseases like bacterial wilt, plants deposit lignin to block pathogen spread [210, 211]. Similarly, cassava deploys cyanogenic glucosides as a fundamental chemical shield. Whitefly feeding provokes the rapid release of these toxic compounds, but the insects continuously detoxify or convert them into harmless forms, highlighting a relentless evolutionary arms race between crops and herbivores [212].

On the abiotic‐stress side, tropical crops rely on highly coordinated antioxidant systems to control excess ROS [213, 214]. During cold stress, sustaining high enzyme activity within the AsA‐GSH cycle can effectively neutralize oxidative damage and preserve tissue quality [215]. Non‐enzymatic components, exemplified by AsA and GSH, form a renewable reducing buffer and, through coupling of the AsA–GSH cycle with enzymes such as APX, sustain H_2_O_2_ detoxification [213]. In mango fruits, AsA and GSH abundance and their stage‐dependent dynamics support a central role for the AsA–GSH cycle in maintaining redox homeostasis during development [216]. Lipid‐soluble antioxidants are likewise critical: papaya accumulates high carotenoid levels that quench singlet oxygen, scavenge free radicals and stabilize membranes, with biosynthesis regulated by both light and stress cues [217, 218].

Notably, antioxidant networks do not operate in isolation, they intersect with photoprotection, heat‐shock responses and osmotic adjustment, forming a dynamic, redundant redox‐regulatory system with feedback capacity [219]. This intersection also extends to biotic stress responses. Pathogen‐induced ROS production, as described above, serves dual roles as both a direct antimicrobial weapon and a signaling molecule that activates secondary metabolite biosynthesis [220]. This integrated system jointly manages the dual roles of ROS as both damaging agents and signaling molecules through multi‐layer coordination, thereby balancing survival, growth and reproduction under compound stresses.

## 5 Molecular and Genetic Adaptation in Tropical Crops

**FIGURE 4:**
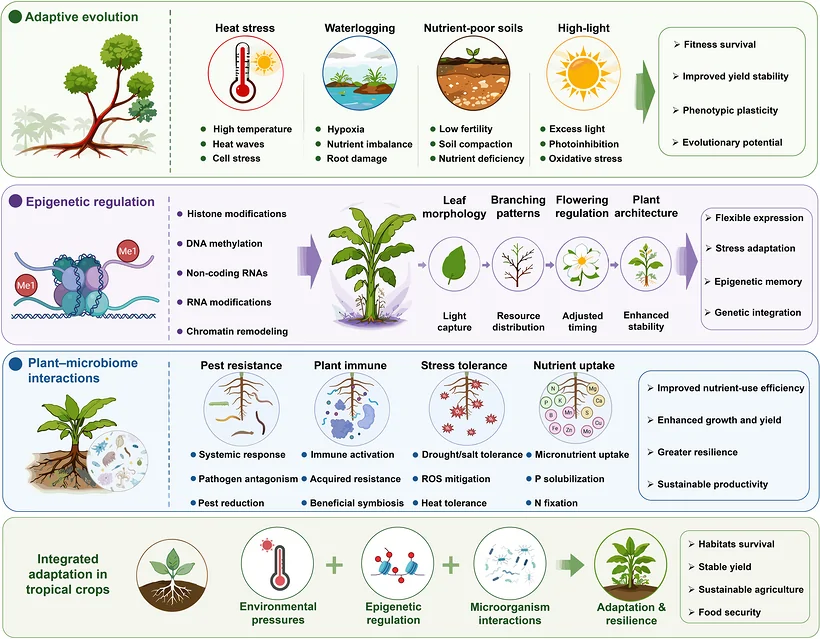
Molecular and genetic adaptations of tropical crops. Integrated framework illustrating adaptive evolution in tropical crops driven by environmental pressures, epigenetic regulation, and plant‐microorganism interactions. Major environmental constraints, including heat, waterlogging, poor soil conditions and strong light, act as selective forces shaping adaptive evolution. These adaptations are mediated by epigenetic regulatory mechanisms, including DNA methylation, histone modifications, chromatin remodeling, non‐coding RNAs, and RNA modifications, which collectively influence plant architecture, leaf shape, branching and flowering. In parallel, interactions with beneficial and pathogenic microorganisms contribute to nutrient absorption, stress resistance, immunity, and pest resistance, forming a coordinated system that underlies phenotypic plasticity and long‐term adaptation in tropical crops. Figure created with BioRender.com.

Extensive genome evolution driven by natural selection and human domestication underpins the complex resilience of tropical crops. Recently, advanced sequencing, multi‐omics, and gene editing have enabled researchers to systematically decode how these plants conquer severe heat, drought, hypoxia and nutrient‐poor soils. This molecular dissection spans sequence variations, epigenetic remodeling, and rewired stress‐signaling networks (Figure 4). Innovative tools like single‐nucleus transcriptomics and chromatin accessibility profiling transform tissue‐specific regulation into concrete evidence to guide precision breeding [221]. The uniqueness of tropical adaptation stems from complex polyploid and highly heterozygous genetic backgrounds. These gene regulatory networks must simultaneously battle compounded threats like intense heat mixed with heavy humidity, blinding light paired with barren soils, or severe drought combined with relentless pests. Consequently, tropical crops demand exceptionally precise, redundant and adaptable signal perception and response mechanisms compared to their temperate counterparts.

### 5.1 Adaptive Evolution of Key Functional Genes

Tropical‐crop genomes harbor numerous key functional genes that, under combined natural selection and domestication, have undergone adaptive evolution, conferring traits essential for stress resilience and high productivity.

Under heat stress, the heat shock protein (HSP) system maintains proteostasis through molecular chaperone activity and constitutes a central defense for cellular thermotolerance. In cassava, the Hsp70 family shows signatures of purifying selection alongside stress‐inducible expression, which provides genetic substrates for adaptation to tropical heat and drought [222]. Small heat shock proteins (sHSPs) form ATP‐independent oligomers, and together with HSP70 and HSP90, they establish a multilayered quality‐control network [223, 224]. HSP expression is precisely regulated by heat shock factors (HSFs).

For waterlogging tolerance, the *Sub1* locus comprises three ethylene response factor genes (*Sub1A*, *Sub1B* and *Sub1C*) [225]. Under submergence‐induced hypoxia, *Sub1A* is rapidly induced. It suppresses gibberellin signaling and ethylene biosynthesis to limit internode elongation and conserve energy, while activating anaerobic metabolism genes such as *ADH1* and *PDC1* [226, 227]. Recent studies indicating that *Sub1A* is embedded within a broader hypoxia‐signaling framework [228]. In parallel, the *Sub1* locus can act alongside the *SNORKEL* (SK) genes, mediating an alternative strategy by promoting ethylene‐driven rapid internode elongation to restore contact with air above the water surface [201]. For abiotic stress adaptation, the same haplotype‐resolved framework identified CBL1 as a gene under selection for cold tolerance during sugarcane domestication and improvement, linking breeder‐favored haplotype discovery with stress‐resilient sugarcane breeding [60].

Under low‐phosphorus stress, tropical crops adapt through coordinated regulation of P mobilization, uptake and internal translocation. In pigeonpea (*Cajanus cajan*), low P induces root secretion of organic acids such as citrate to chelate Fe/Al and mobilize fixed P, accompanied by upregulation of the PHT1‐family transporter *PHT1;4* to enhance uptake. Concurrently, root morphology and associated physiological traits exhibit plastic changes, forming an integrated network from signal perception to morphological and physiological responses [229, 230].

Interestingly, the evolution and regulation of photosynthetic‐efficiency genes underlie the high productivity of many tropical crops. In tropical grasses, C4 photosynthesis (e.g., sugarcane, sorghum) relies on spatial division of labor and coordinated enzyme expression between mesophyll and bundle sheath cells [162]. In sugarcane, high biomass and sugar accumulation arise from tight coupling among carbon‐fixation efficiency, carbon partitioning and stem sink strength. However, sucrose transport and storage, which ultimately determine yield and sucrose deposition from leaf sources to stem sinks, are jointly controlled by multiple gene classes [231, 232]. Recent advances reveal that sugarcane C4 photosynthesis is controlled by a gene regulatory network, the decarboxylase C4‐specific nicotinamide adenine dinucleotide phosphate‐malic enzyme (C4NADP‐ME) is regulated by abscisic acid insensitive 5 (ABI5) via a G‐box motif [233], while miRNAs indirectly regulate C4 genes via transcription factors like SsHAM3a, adding post‐transcriptional control along the leaf gradient [233]. Sucrose phosphate synthase/sucrose synthase (SPS/SS) enzymes, transporters and TFs provid candidate gene sets for molecular design breeding [232]. In sorghum, there are clear cell‐type preferences for genes involved in photorespiration and the C4 cycle [234]. Many C4 key‐enzyme genes arose from C3 ancestral genes through duplication and functional divergence, accompanied by recruitment and remodeling of promoter sequences, revealing the evolutionary origins of cell‐type–specific regulatory networks [235].

### 5.2 Molecular Mechanisms of Heat Stress Responses in Tropical Crops

In tropical crops, heat stress often occurs together with strong irradiance, drought and high humidity [236]. Therefore, heat tolerance in these crops should be interpreted as a coordinated response that maintains protein homeostasis, redox balance, photosynthetic stability and fruit quality rather than as a generalized heat shock response.

At the protein level, HSP and HSF systems form the first protective layer. Overexpression of *EaHSP70* derived from the related wild species *Erianthus arundinaceus* in sugarcane significantly enhances the cell membrane thermal stability, relative water content, gas exchange parameters, chlorophyll content and photosynthetic efficiency of transgenic plants, while simultaneously upregulating the expression of multiple stress‐responsive genes [237]. In cassava, genome wide analysis identified 22 Hsp70 genes with stress related cis elements and tissue specific or stress inducible expression, suggesting that Hsp70 family expansion provides a molecular basis for adaptation to tropical heat and drought [222]. In banana, acquired thermotolerance is associated with the induction of heat shock factors, heat shock proteins, stress associated proteins, ROS scavenging enzymes, fatty acid metabolism and protein modification pathways [238]. Together, these responses maintain protein folding capacity and limit the accumulation of damaged proteins under acute heat exposure.

At the redox and metabolic levels, thermotolerance is closely linked to ROS control. In cassava, high temperature responses differ between vascular and mesophyll tissues, indicating tissue‐specific regulation of heat stress perception and response [239]. Physiological studies further show that heat challenged cassava can adjust stomatal behavior, accumulate soluble sugars and proline and activate antioxidant enzymes such as SOD and APX to reduce oxidative damage [176]. In banana, the heat responsive transcriptional activator MaHSF11 directly activates MaFLS1 and MaF3'5'H1 and promotes myricetin accumulation, linking HSF signaling with flavonol mediated ROS buffering [240]. This connection is important in tropical environments, where heat usually occurs together with strong light and oxidative pressure.

At the chloroplast and photosynthetic levels, tropical crops maintain carbon assimilation through structural and regulatory changes in photosynthetic pathways. Cultivated cassava shows C3 to C4 intermediate photosynthetic features and high photosynthetic efficiency compared with wild relatives [241]. *MeNADP‐ME3* is regulated by light intensity and its promoter Indel sites are predicted to be bound by MeYABBY1, forming a regulatory connection with photosynthesis related genes [242, 243]. These findings suggest that cassava photosynthetic adaptation may buffer the combined pressure of heat, high light and drought. In sugarcane, ABI5 mediated regulation of C4NADP‐ME and miRNA mediated control through *SsHAM3a* connect C4 gene regulation with heat and light adaptation [233]. These examples highlight chloroplast centered photosynthetic stability as a distinctive feature of tropical crop thermotolerance.

**FIGURE 5:**
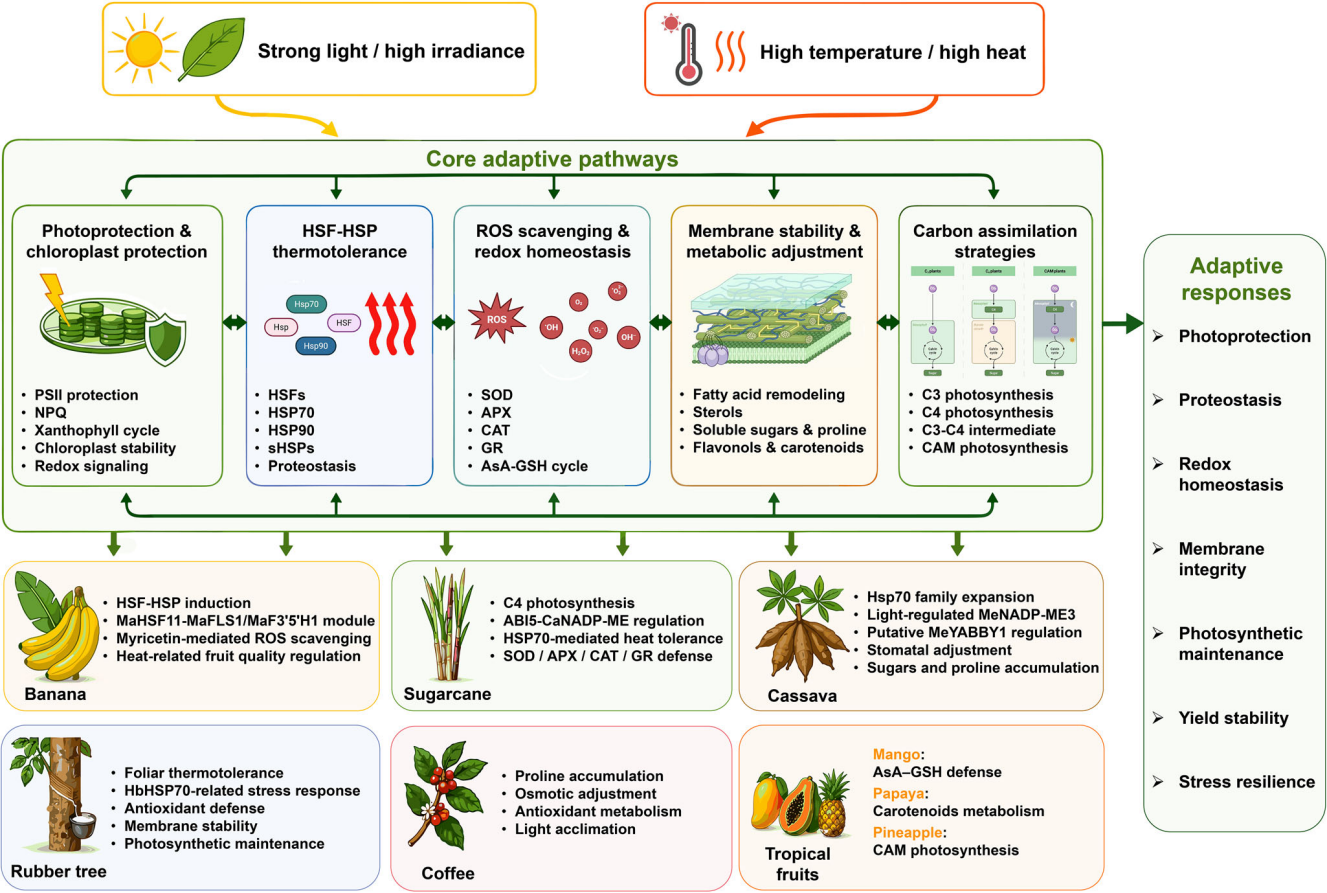
High‐light adaptation and thermotolerance pathways in representative tropical crops. Schematic overview of conserved and crop‐specific pathways associated with high‐light adaptation and heat tolerance in tropical crops. Adaptive responses are summarized across five major modules, including light/chloroplast protection, HSF–HSP‐mediated thermotolerance, ROS detoxification and redox homeostasis, membrane and metabolic adjustment, and photosynthetic carbon‐assimilation strategies. Representative crop examples include banana, sugarcane, cassava, rubber tree, coffee and tropical fruits, highlighting their coordinated contributions to photoprotection, protein stability, redox balance, membrane integrity, sustained photosynthesis and yield or quality resilience under tropical high‐light and heat‐stress conditions. Figure created with BioRender.com.

Heat also affects fruit quality in tropical horticultural crops. In banana, the MaASR3 and MaHDT1 regulatory complex suppresses *MaNIP1* and modulates high temperature inhibited chlorophyll degradation during fruit ripening [244]. By contrast, direct evidence for stable heat stress memory in tropical crops remains limited. Overall, high‐light adaptation and thermotolerance in tropical crops are coordinated by several conserved modules, including light/chloroplast protection, HSF–HSP‐mediated proteostasis, ROS detoxification via antioxidant enzymes and the AsA–GSH cycle, membrane and metabolic adjustment and photosynthetic carbon‐assimilation strategies. Representative examples from banana, sugarcane, cassava, rubber tree, coffee, and tropical fruits illustrate how these pathways jointly support photoprotection, protein stability, redox balance, membrane integrity, sustained photosynthesis and yield or quality resilience under tropical high‐light and heat‐stress conditions (Figure 5).

### 5.3 Epigenetic Regulation and Genetic Diversity

Beyond DNA sequence variation, epigenetic regulation, together with population‐level genetic diversity, is a critical foundation for both rapid adaptation and long‐term evolution in tropical crops. As reported, epigenetic mechanisms can rapidly reprogram gene expression without altering DNA sequences and generate stress memory, thereby enhancing response efficiency and maintenance of homeostasis upon recurrent stress exposure [245, 246, 247].

In sugarcane, the tissue‐specific methylation patterns are tightly coupled to tissue‐specific gene expression, indicating that within an extremely large, repeat‐rich genome, DNA methylation mediates both transposon silencing and fine‐scale gene regulation, shaping hormone signaling and expression of stress‐resistance genes [248, 249]. At the non‐coding RNA layer, lncRNAs and miRNAs modulate immunity and metabolic reprogramming in sugarcane response to smut infection by targeting defense and signaling genes [250].

During fruit ripening, dynamic changes in DNA methylation occur in banana under pathogen stress. These changes are coordinated with expression of defense‐ and ripening‐related genes [251]. At the histone‐modification level, banana ripening involves both Jumonji‐mediated histone demethylation and TF–mediated recruitment of HDAC‐containing repressor complexes to finely tune ethylene responses and ripening‐gene expression [252, 253]. In extreme tropical ecosystems, studies of *Avicennia marina* and *Rhizophora apiculata* reveal pronounced interspecific differences under high UV‐B conditions in transposon silencing, methylation states and transcriptional responses, accompanied by strengthened protective metabolic pathways such as flavonoid biosynthesis [254, 255]. In cassava, histone deacetylase 9 (HDA9) regulates melatonin accumulation by modulating histone deacetylation at melatonin‐biosynthesis genes, thereby altering resistance to bacterial blight [256]. More recently, targeted methylation of effector‐binding sites in promoters of susceptibility genes blocks effector binding and prevents their induced expression, significantly reducing disease symptoms and illustrating the feasibility of epigenome editing for disease‐resistance breeding in tropical crops [257]. Collectively, epigenetic memory enables faster and stronger responses to recurrent stresses [247, 258].

In addition, genetic diversity and evolution remain the ultimate sources of tropical‐crop adaptability. Harnessing wild relatives such as *Fusarium wilt* resistance genes from wild banana to generate novel germplasm is a key strategy for broadening the genetic base of modern cultivars and enhancing environmental resilience [259, 260].

### 5.4 Interactions With Symbiotic Microorganisms

Mutualistic associations between tropical crops and soil or endophytic microorganisms constitute a major eco–molecular strategy for coping with nutrient‐poor soils and high biotic‐stress pressure. They are governed by conserved modules of host recognition–signal transduction nutritional and immune remodeling, while displaying species‐specific regulatory features across crops [261]. This symbiotic relationship has evolved from an optional benefit to a survival necessity, which is one of the key features that distinguish tropical crops from temperate crops.

Establishment of arbuscular mycorrhizal (AM) symbiosis begins with plant recognition of fungal Myc factors, activation of the common symbiosis pathway, induction of nuclear calcium oscillations, and downstream transcriptional reprogramming. This cascade enables fungal entry and arbuscule formation, allowing reciprocal exchange in which plants supply carbon and fungi provide mineral nutrients [262]. In banana, the genome of the root‐derived endophyte *Bacillus amyloliquefaciens* encodes multiple biosynthetic gene clusters for antimicrobial lipopeptides (surfactin, iturin) and polyketides (difficidin), which directly inhibit the *Fusarium wilt* pathogen *Fusarium oxysporum* f. sp. *cubense* (Foc) [263]. From the host‐response perspective, disease‐resistant banana can rapidly activate respiratory metabolism and defense signaling during early infection [264], and it also mobilizes defense pathways and stress networks earlier during TR4 invasion to limit pathogen spread within vascular tissues [265]. Beyond disease resistance, broad‐host‐range endophytic fungi enhance tolerance to drought and salinity by secreting effectors or signaling molecules that regulate host antioxidant systems and ABA‐signaling gene expression, thereby improving survival and growth under stress [261]. In trifoliate orange (*Poncirus trifoliata*), colonization by *Piriformospora indica* increases activities of antioxidant enzymes and is accompanied by shifts in fatty‐acid composition and unsaturation indices, reducing ROS accumulation and lipid peroxidation damage [266]. In sugarcane, smut is a major disease, and multiple *Trichoderma* isolates have been screened for antagonistic activity against this pathogen [267]. Metabolites from several *Bacillus* species can effectively inhibit or disrupt growth and reproduction of the sugarcane smut fungus [268]. Studies using sugarcane rhizosphere actinomycetes show that *Streptomyces griseorubiginosus* BTU6 not only demonstrates antagonistic activity but also enhances smut resistance by regulating host defense‐enzyme activities and accumulation of secondary‐metabolite–related compounds. During pathogen invasion, BTU6 induces sugarcane production of chitinases, β‐1,3‐glucanases and multiple secondary metabolites, thereby strengthening resistance [266, 269].

Overall, symbiotic interactions in tropical crops converge on three intertwined gain pathways: First, AM symbiosis enhances mineral nutrient acquisition and remodels root signaling networks [262], Second, rhizosphere and endophytic bacteria suppress disease through antagonistic metabolites and immune induction [263, 264, 265], and Third, broad‐host‐range endophytic fungi improve stress tolerance by regulating antioxidant capacity and membrane‐lipid and osmotic homeostasis [242, 261, 266].

## 6 Ecological‐Scale Adaptation in Tropical Crops

**FIGURE 6:**
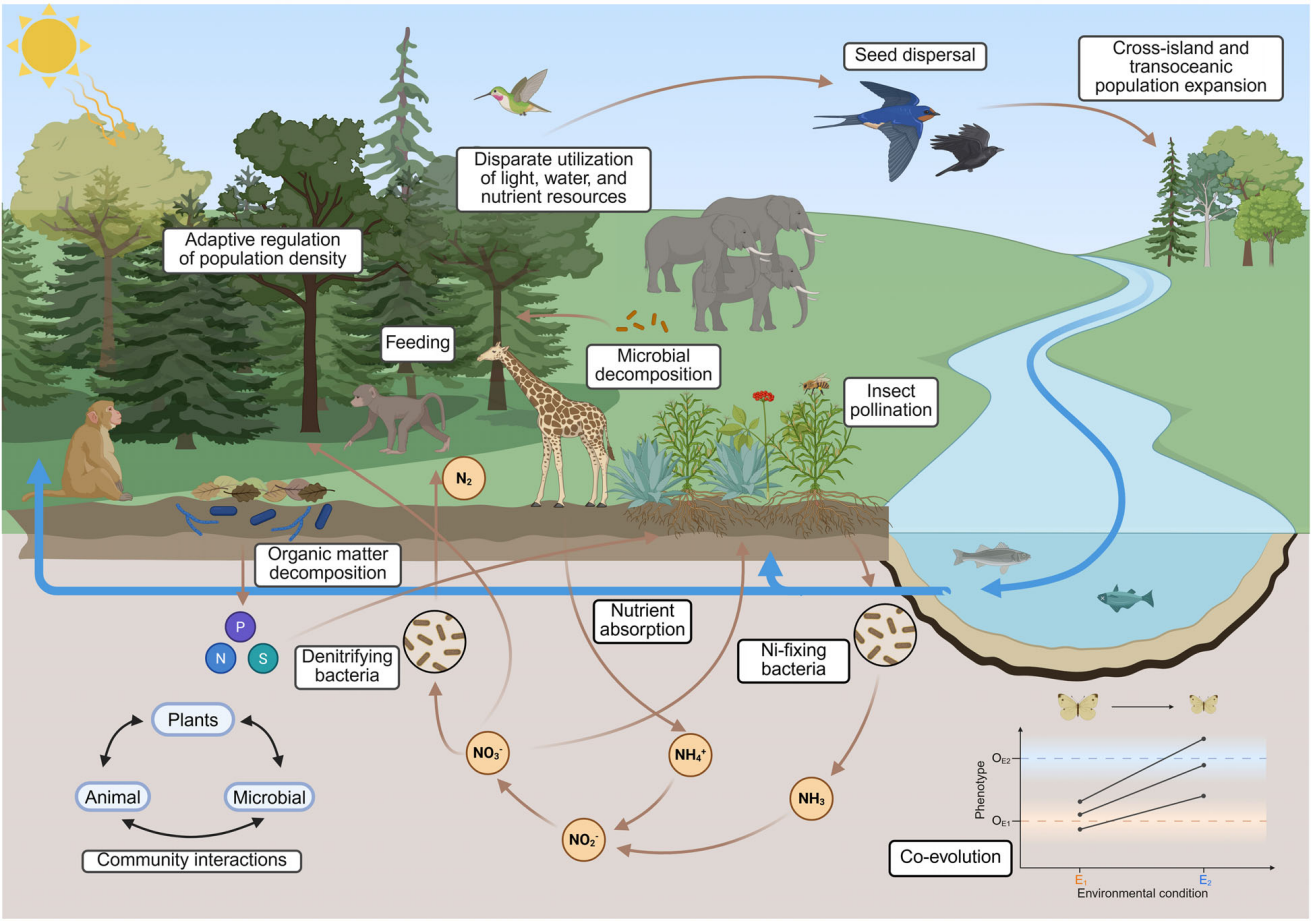
Ecological adaptations of tropical crops. Schematic illustration of multi‐trophic community interactions and biogeochemical cycling linking biodiversity, ecosystem functions and population processes. Plants, animals and microorganisms jointly regulate organic matter decomposition, microbial mineralization and nutrient transformations, including nitrogen fixation, nitrification and denitrification, thereby shaping nutrient availability and plant nutrient absorption. Aboveground interactions such as herbivory and feeding, insect pollination and seed dispersal contribute to plant reproduction and community assembly, while resource partitioning promotes the disparate utilization of light, water and nutrient resources. These coupled processes influence adaptive regulation of population density and facilitate population expansion across islands and transoceanic dispersal. The framework further highlights co‐evolutionary dynamics between organisms under contrasting environmental conditions. Figure created with BioRender.com.

Tropical crops, most probably through both natural selection and human domestication, have evolved morphological and physiological traits as well as ecological strategies that finely regulate life‐history characteristics to cope with frequent disturbance, intense competition, and strong spatiotemporal heterogeneity of resources in tropical environments. These strategies reflect a trade‐off between rapid expansion and opportunistic niche occupation and the maintenance of genetic diversity and long‐term stable renewal, providing an ecological foundation for the sustainability of tropical agricultural systems. Unlike temperate agricultural ecosystems, tropical agricultural ecosystems face higher biodiversity, more intense interspecific competition, stronger spatiotemporal resource heterogeneity and more frequent natural disturbances (such as typhoons and heavy rain), which means that the ecological adaptation of tropical crops must be based on complex multispecies interactions and dynamic equilibrium (Figure 6).

### 6.1 Population Strategies

Population persistence and spread in tropical crops depend on the coordinated functioning of reproduction, dispersal, and density regulation. In annual or short‐lived crops, high reproductive rates buffer populations against environmental variability. By contrast, perennial or clonally propagated crops more often employ a dual‐channel strategy in which asexual reproduction ensures efficient stand establishment, whereas sexual reproduction maintains genetic diversity and adaptive potential. Cassava, for example, is propagated mainly through stem cuttings, yet its sexual reproductive system remains functional. Wild relatives can produce large seed numbers, enabling rapid post‐disturbance colonization while sustaining genetic diversity [270, 271]. This asexual reproduction as the main method, sexual reproduction as a supplement strategy is an adaptive double insurance against frequent disturbances in tropical regions.

Seed dispersal determines dispersal distance, establishment success, and gene flow intensity. Wild and semi‐wild populations of understory fruit trees and agroforestry crops commonly achieve cross‐patch dispersal via animals. In cocoa, animal‐mediated dispersal operates in natural systems, while in managed landscapes, human activities further enhance long‐distance dispersal [272]. In agroforestry mosaics, secondary dispersal by wildlife can occur, chimpanzees consuming cocoa fruits and transporting or excreting seeds, suggesting that primates can facilitate spatial spread and reduce near‐maternal competition [273, 274, 275].

Density regulation maintains population stability and optimizes resource use. At high density, competition for light, water and nutrients induces self‐thinning, returning populations toward carrying capacity. In rubber plantations, for example, the number of tappable trees and tapping density fluctuate with extreme weather and wind or cold damage, optimizing planting patterns, management practices and stress‐resilience breeding can increase the retention of tappable trees per unit area and stabilize dry‐rubber yield [276]. From above, population strategies in tropical crops are not merely high reproduction/fast dispersal, but rather integrated systems that balance rapid establishment with long‐term adaptation under frequent disturbance and intense competition.

### 6.2 Community Interactions

At the community scale, tropical crops are embedded in complex interaction networks with plants, animals, and microorganisms. These networks shape key ecosystem services, including competition, pollination, seed dispersal and pest and disease suppression, and thereby determine system stability and productivity. The extremely high biodiversity in tropical regions makes these interaction networks exceptionally complex, often exhibiting high levels of specialization and functional irreplaceability, which are rare in temperate ecosystems.

Allelopathy exemplifies chemically mediated competition. Aqueous extracts from leaves, stems, and roots of coffee inhibit germination and radicle elongation in receiver plants, with candidate allelochemicals including caffeine, chlorogenic acid, ferulic acid and *p*‐coumaric acid [277]. Similarly, alkaloids and phenolic acids in litter and root exudates suppress weed germination and early growth in coffee, reducing competitive pressure for resources in understory or agroforestry systems [278].

Co‐evolution is evident in interactions with pollinators and dispersers. *Vanilla planifolia* is pollinated primarily in its native range by specific orchid bees (*Euglossa* spp.) and sometimes hummingbirds [279, 280]. On the other hand, cocoa relies largely on small insects such as midges and floral volatiles may regulate pollinator behavior through scent blends, providing mechanistic targets for improving fruit set [281]. In major oil palm production regions, pollination depends heavily on weevils. Their populations are constrained by rainfall and humidity, pesticide exposure, natural enemies and landscape structure, making pollination stability a key ecological determinant of yield variability [150]. For some palms and large fleshy‐fruited plants, dispersal by medium‐to‐large mammals is functionally irreplaceable. For instance, lowland tapirs markedly extend dispersal distances for large palm seeds, thereby influencing gene flow and recruitment sites [282]. Many crops demonstrate preferences for or even obligate dependence on specific pollinators or dispersers, declines of key animals driven by habitat fragmentation, pesticide pressure or climatic anomalies can cause systemic losses of pollination and dispersal services. Consequently, conserving and restoring habitats for these animal partners is an ecological foundation for sustaining crop productivity and genetic diversity [283].

### 6.3 Niche Differentiation

In highly diverse tropical systems, niche differentiation reduces competition by partitioning resource use across time, space or functional modes. In cocoa agroforestry, upper‐canopy and shade trees buffer excessive radiation, while shade‐tolerant cocoa exploits transmitted and diffuse light and benefits from a buffered microclimate. Shade‐tree composition and pruning regimes modify throughfall and microclimatic conditions, thereby shaping the growth environment [284, 285, 286]. Distinct canopy architectures alter shading intensity and spatial heterogeneity, shaping trade‐offs between ecosystem services and yield [287]. Moreover, system type and environmental context can influence bean maturation and post‐harvest conditions, thereby modifying the sensory profile of dark chocolate [288]. This stratified use of light resources is a sophisticated ecological adaptation to the intense radiation and high‐temperature stress in tropical regions.

Belowground niche differentiation is expressed through complementarity in rooting depth and nutrient acquisition strategies. Deep‐rooted trees access deep soil water and leached nutrients and return them to surface layers via litter, forming a nutrient pump/safety‐net mechanism that enhances resilience in infertile, strongly leached soils [289]. Root stratification among crops—such as deep‐rooted coconut and mango versus shallow‐rooted pineapple and peanut—enables complementary use of water and nutrients [290]. Legumes supply nitrogen through biological fixation, whereas cassava occupies a distinct “nutritional niche” by mobilizing fixed nutrients through rhizosphere acidification and organic‐acid ligands [291]. In cocoa systems, vertical and horizontal complementarity between cocoa roots and leguminous shade trees such as *Inga edulis* is also evident [292]. Rhizosphere studies further show that cassava enhances phosphorus dissolution and multi‐element uptake by releasing protons and organic acids, with these effects modulated by phosphate‐mineral form and solubility [101].

Resource complementarity frequently increases land equivalent ratio (LER) above 1, indicating that total output per unit land exceeds the summed outputs of component monocultures [293, 294]. Increasing diversity also enhances ecological stability. Complex community structures strengthen natural‐enemy pest control, improve soil structure, carbon sequestration water retention and stabilize microclimates [295, 296, 297]. Compared with monocultures, well‐structured agroforestry systems often exhibit higher soil microbial diversity and activity [298] and greater soil carbon‐sink potential [299]. Such systems can also markedly reshape microbial community composition, as shown in tropical rubber intercropping and cocoa agroforestry, where microbial diversity and activity are consistently enhanced [298, 300].

Taken together, ecological adaptation in tropical crops—from population strategies to community interactions and niche differentiation—forms a multilevel, networked system. Designing and managing production systems around these mechanisms is essential for building tropical agricultural landscapes that combine high productivity, strong climate resilience and environmental sustainability.

## 7 Emerging Challenges

Despite their multi‐layered adaptive strategies, tropical crops now face systemic pressures from climate change and human activities, most notably the rising frequency of compound extreme events that simultaneously destabilize production and degrade product quality. These challenges present a unique cumulative amplification effect in tropical regions: the tropical baseline characteristics such as high temperature, high humidity, strong winds and nutrient‐poor soils interact with extreme events caused by global climate change, posing an unprecedented systemic threat to tropical crop production.

### 7.1 Compound Risks of Heat, Drought and Extreme Rainfall/Storms

Climate change–driven compound extremes constitute the most immediate and rapidly intensifying threat. Sustained warming can push growing conditions beyond physiological optima and induce concurrent yield–quality declines by disrupting flowering, fruit and seed set, grain filling or organ bulking and quality‐related metabolic pathways. In sugarcane, combined heat and drought constrain photosynthesis, disrupt sucrose accumulation and transport and amplify yield variability [301]. Similarly, oil palm suffers from pronounced yield sensitivity to climate anomalies, particularly heat and drought [302].

In addition to heat and drought, extreme storms and typhoons represent a dominant physical hazard to banana [303]. To address this unique issue, executable solutions include: (1) breeding dwarf compact varieties to lower the plant's center of gravity, (2) optimizing planting density and row spacing configurations, using a “wide‐narrow row” planting pattern to enhance wind resistance, (3) developing smart support systems, using biodegradable materials to construct field supports, (4) establishing a typhoon early warning and emergency response mechanism, performing moderate pruning and reinforcement before a typhoon arrives. For sugarcane, in response to combined heat and drought stress, it is recommended to breed genotypes with an efficient C4 photosynthetic system, strong root penetration, and high osmotic regulation ability, supported by drip irrigation and plastic mulch technologies.

Cassava is often described as “climate‐resilient,” yet warming and increasingly unstable rainfall regimes can still shift yields systematically. Moreover, genotypic differences in drought‐timing sensitivity strongly affect the stability of storage‐root dry matter and starch yield [304]. Coffee is even more temperature‐ and water‐sensitive: its approximate optimal mean annual temperature range is ∼18°C–22°C, and exceedance elevates risks of abnormal flowering and accelerated ripening, with downstream effects on flavor stability [305, 306]. The flowering‐to‐fruit development phase is acutely sensitive to temperature, water availability and radiation, phenological disruption readily causes flower and fruit drop and uneven bean development [307].

Drought is increasingly translating physiological differences directly into agronomic performance gaps [308]. In major production regions, recurrent severe drought has already reduced photosynthetic efficiency, intensified wilting and caused substantial yield losses. Concurrently, extreme storms (tropical cyclones and typhoons) pose a persistently rising risk of physical damage to tall perennial crops such as oil palm, banana and rubber.

### 7.2 Dual Erosion of Genetic Foundations and Biosecurity

Habitat destruction and biological invasions are simultaneously undermining tropical agricultural resilience by eroding genetic foundations and weakening biosecurity. On one front, modern breeding in several key crops is constrained by narrow parental pools and complex genetic architectures, slowing responses to emerging pests, pathogens and climate extremes. On the other, accelerated trans‐regional spread and rapid evolution of pests and pathogens are imposing escalating systemic risks on quarantine and production systems.

Sugarcane improvement has relied to a degree on a limited set of parents within an extremely complex polyploid genomic background, raising barriers to genetic dissection and rapid genetic gain and constraining responses to novel pests, diseases and climate shocks [309]. Banana systems exemplify this vulnerability through the spread of *Fusarium wilt* Race 4. In recent years, its dissemination has accelerated and is now regarded as a systemic threat to global banana production [310, 311]. TR4 is especially destructive to the dominant Cavendish subgroup, and the genetic uniformity of major commercial cultivars further amplifies industry‐wide risk [311, 312]. Mechanistically, accessory genes, nitric‐oxide biosynthesis and related factors have been implicated in TR4 virulence, indicating deep genetic and physiological bases for the difficulty of effective control [313]. To address banana TR4 disease, a comprehensive control system should be established based on rapid molecular diagnostic field detection systems, combined with the promotion of resistant varieties, soil disinfection and crop rotation systems.

Simultaneously, tropical deforestation and land conversion are fragmenting and eliminating habitats of crop wild relatives, steadily eroding the genetic “insurance vault” needed for future crop improvement. Multiple subspecies of wild banana, for example, are key sources of disease‐ and stress‐resistance alleles [314]. In cassava, bio‐security risks are tightly coupled to vegetative propagation systems, enabling viruses to spread efficiently through planting material and imposing chronic management burdens [315]. In oil palm, basal stem rot caused by *Ganoderma boninense* represents a major production bottleneck in Southeast Asian plantations; resistance is largely quantitative, implying that long‐term improvement requires larger breeding populations and stronger molecular tools [316]. In coffee, erosion of genetic foundations is driven primarily by contraction of wild populations and suitable habitats. It is often associated with compound heat–drought stress, making future improvement highly dependent on wild germplasm and diversified cultivation systems to buffer climatic and biotic pressures [317].

### 7.3 A “Slow‐Variable” Crisis Under Infertile Tropical Soils

Soil degradation constitutes a fundamental long‐term constraint on tropical crop productivity. To address the unique issues of tropical soil acidification, nutrient deficiency and organic matter loss, executable solutions include: (1) promoting cover crops and green manure rotation systems, (2) applying biochar amendments to produce biochar, improving soil pH, cation exchange capacity and water retention, (3) implementing precision fertilization techniques, reducing nutrient loss and non‐point source pollution, (4) establishing agroforestry systems, pairing deep‐rooted trees with shallow‐rooted crops to achieve nutrient pumping and soil erosion control, (5) developing genotypes that are tolerant to aluminum toxicity and efficient in low‐phosphorus environments, combined with root exudate regulation techniques to improve nutrient use efficiency. In sugarcane, once water and fertility supplies become decoupled, extreme drought years more readily trigger sharp yield losses and sucrose‐quality instability [301]. In intensive oil palm systems in Southeast Asia and elsewhere, prolonged monoculture, heavy fertilizer use and suboptimal soil management drive severe soil acidification, rapid declines in organic matter, and strong nutrient imbalance directly undermining long‐term yields and palm‐oil quality [318]. In banana, waterlogging, lodging, and root injury caused by heavy rainfall and typhoons are not merely short‐term losses, by damaging root health and destabilizing soil microbial communities, they elevate subsequent soil‐borne disease pressure [303].

In traditional slash‐and‐burn systems, population pressure has sharply shortened fallow periods, leaving insufficient time for soil fertility recovery. Consequently, soil nutrient are persistently depleted and soil structure degrades [319, 320]. This severely constrains sustainable production of staples such as cassava and further accelerates erosion and land degradation [321].

## 8 Future Research Directions for Tropical Crop Adaptation

A major unresolved question is how tropical crops perceive and integrate compound stresses that commonly occur together in tropical environments, such as heat, humidity, intense radiation, alternating drought and flooding, acidic nutrient poor soils, and persistent biotic pressure. Future work should therefore shift from single stress descriptions toward integrated frameworks that connect stress signaling, adaptive alleles, physiological traits and field level performance. Wild germplasm, pan‐genome resources, multi‐omics approaches, and ecological design will be essential for identifying selectable traits and developing cultivar and cultivation strategies that are both deployable and resilient under tropical production conditions.

### 8.1 Multi‐Factor, Multi‐Level Dissection of Synergistic Multi‐Stress Adaptation

In the field, stresses rarely occur in isolation but instead act simultaneously or sequentially (e.g., drought × heat, high light × heat, flooding × hypoxia, low P × metal toxicity), often producing nonlinear yield losses that exceed the sum of individual effects. When drought and heat co‐occur, multiple damage pathways stack to drive yield losses far beyond additive expectations [3, 4, 5, 322]. In rice and maize exposed to combinations of heat, salinity, low phosphorus, cadmium, oxidants and other factors, growth declines progressively as the number of concurrent stressors increases [323].

For tropical regions, it is recommended to prioritize focusing on more tropical‐specific multi‐stress, rather than just the common drought × heat stresses seen in temperate crops. For example: (1) high temperature × high humidity (night‐time high temperature/high humidity increases respiratory consumption, obstructs pollination and fertilization, and enhances pathogen proliferation), (2) intense light × high temperature × intermittent drought (causing combined light inhibition and thermal/oxidative damage), (3) monsoon region flooding × hypoxia × high temperature (root suffocation and pathogen synergy), (4) acidic, nutrient‐poor soils with low phosphorus × aluminum toxicity/manganese toxicity × leaching (limiting root access and nutrient utilization), (5) year‐round pest, disease and weed pressure × climate variability (faster population dynamics and resistance evolution). These frequently co‐occurring combinations should become priority targets for constructing multi‐factor platforms for tropical crops and for phenotype–omics analysis.

Therefore, future work should focus on an integrated framework that links multi‐stress phenotyping, multi‐omics, and pan‐genome or super‐pangenome resources to identify adaptive alleles, presence‐absence variations, structural variants and regulatory networks underlying tropical crop resilience [58, 324, 325, 326, 327, 328]. This streamlined framework can connect combined‐stress responses with deployable targets for stress‐resilient breeding.

### 8.2 Translating Mechanistic Targets Into Stress‐Resilient Cultivar Portfolios

Genomic resources including reference genome assemblies, pan‐genomes, and marker‐trait associations directly enable genomics‐assisted breeding (GAB) strategies. To further support the practical use of genomic and digital resources in tropical crop improvement, we summarized representative public databases in Table S1. These resources, including general tropical crop databases and crop‐specific platforms, provide accessible information on germplasm, genomes, variants, gene expression, phenotypes, omics datasets and breeding‐related tools. Moreover, the integration of artificial intelligence, machine learning and high‐throughput phenotyping with public databases is expected to accelerate target discovery, genomic prediction and climate‐resilient cultivar development in tropical crops. GAB integrates marker‐assisted selection (MAS) for major‐effect loci and genomic selection (GS) for polygenic traits, thereby accelerating genetic gain and shortening breeding cycles in tropical crops [329]. For polyploid crops such as sugarcane, GS models that incorporate allele dosage effects can improve prediction accuracy [23, 24, 59, 60, 309]. For clonally propagated crops such as cassava and banana, MAS and GS enable early selection before field phenotyping, reducing generation time [65, 322, 330]. These GAB strategies, when coupled with speed breeding protocols, offer a realistic pathway to develop climate‐resilient tropical crop varieties efficiently.

Molecular design breeding advances crop improvement from experience‐based selection toward predictable, editable precision design. Genomic selection uses genome‐wide markers to predict complex polygenic traits (e.g., drought tolerance, yield) early in the breeding cycle, accelerating genetic gain and shortening breeding timelines [331]. Implementation priorities, however, differ among tropical crops. For the polyploid nature of sugarcane, integrating polymorphic markers such as SNPs, copy number variations (CNVs), and SVs, and developing GS statistical models that incorporate allele dosage effects [332]. For cassava, taking advantage of its shorter growing cycle to establish a rapid‐cycle GS system, shortening the breeding cycle to 3–4 years [330]. For oil palm GS, integrating whole‐genome sequence data with pedigree information to develop multi‐trait selection indices suitable for species with long generation intervals [333, 334]. Due to the triploid nature of banana limiting recombination, focus on developing haplotype‐based GS models, combined with genome‐assisted backcross designs, to efficiently introgress resistance genes. Additionally, specific training population design standards for tropical crops need to be established to optimize the balance between prediction accuracy and the maintenance of genetic diversity.

Gene editing technologies enable precise gene knockout, replacement, or regulatory tuning. In tropical crops, (1) using CRISPR/Cas9 technology to edit the *SPS* and *SS* gene families in sugarcane to optimize sucrose transport efficiency, developing allele‐specific editing strategies for polyploid sugarcane to overcome technical barriers caused by gene dosage effects, (2) using single‐base editing technology to modify the promoter region of the *HDA9* gene in cassava to enhance disease resistance, (3) using Prime Editing strategies to precisely replace key genes in the ethylene signaling pathway of banana to regulate fruit ripening, (4) establishing protoplast regeneration and genetic transformation systems for tropical crops to overcome genotype‐dependent limitations. These technological paths need to be combined with high‐throughput phenomics platforms to build precise genotype‐phenotype‐environment association models.

Equally indispensable is systematic exploitation of wild relatives. (1) Establishing in situ and ex situ conservation networks for wild relatives of tropical crops, focusing on the collection and preservation of marginal populations with extreme environmental adaptability. (2) Developing genome selection‐based gene introgression optimization algorithms to maximize the retention of wild alleles while minimizing linkage drag. (3) Establishing a phenomics database for wild germplasm resources to systematically analyze the genetic basis of adaptive traits. Through introgression breeding or editing‐based strategies, transferring superior alleles that have been lost or remain underutilized in cultivated germplasm is a direct route to rapidly broaden genetic bases and achieve step‐change gains [7, 335].

### 8.3 Optimizing Ecological Fit of Tropical Agroforestry for Sustainable Management

Enhancing ecosystem‐scale resilience requires moving agroforestry from qualitative description to quantitative analysis and model‐based optimization. In “rubber–cocoa” systems, for example, rubber canopies reduce understory temperature and evaporative demand, filter excessive radiation while increasing diffuse light, and thereby alleviate dry‐season photoinhibition and improve cocoa micro‐environments [285, 286, 287, 336]. At the design level, species composition, canopy structure, planting density and spatial arrangement should be optimized jointly. Reconfiguring landscape structure and habitat arrangement can enhance resilience and service provision [337].

At the management level, variable‐rate fertilization guided by soil and plant diagnostics can reduce nitrogen and phosphorus losses while increasing fertilizer‐use efficiency and has proven effective in crops such as pineapple and oil palm [338]. In cocoa and coffee, agroforestry systems that retain or plant shade trees buffer heat and water stress, enhance carbon and nutrient cycling via litter and root activity, and maintain habitats for pollinators and natural enemies—thereby coupling production with ecosystem services [339]. In seasonally dry regions, drip irrigation or micro‐sprinklers combined with cover crops or mulching/film coverage can reduce evaporation and help manage increasingly unstable rainfall [340]. Accordingly, future work should integrate process‐based models with optimization algorithms to identify optimal solutions across “yield–quality–resilience–ecosystem services” under given climate, soil and management constraints, and develop replicable configuration templates tailored to different tropical regions.

Finally, systematic conservation and utilization of crop wild relatives represent a strategic safeguard for long‐term breeding. Conservation strategies should balance in situ and *ex situ* approaches—for example, establishing protected sites for wild rice, wild coffee and other key taxa to preserve evolutionary potential and ecological interactions [341, 342]. In parallel, deep phenotyping and whole‐genome resequencing of core germplasm should be strengthened to build genotype–phenotype–environment association databases, which, combined with genomics and gene editing, can be leveraged to precisely identify elite alleles for deployment [343].

## 9 Technical Pathways for Cross Species Transfer of Tropical Adaptive Traits

Tropical crops have evolved diverse adaptive traits under long‐term exposure to compound stresses, including heat, humidity, intense light, seasonal drought and flooding, acidic nutrient‐poor soils and persistent biotic pressure. These traits provide a valuable source of transferable mechanisms for improving crop resilience in both tropical regions and marginal environments. Here, we propose an integrated cross‐species transfer framework that converts evolutionary and ecological insights into practical strategies through genetic introgression, functional validation, regulatory optimization, microbiome management, agronomic integration and field assessment of yield, stability and cost.

### 9.1 Identification and Deconstruction of Transferable Adaptive Modules

Cross‐species transfer abandons the search for single miracle genes and instead conceptualizes tropical advantages as distinct modules with clear boundaries. A module represents specific measurable phenotypes and their driving mechanistic nodes that actively secure survival in harsh tropical environments. These robust modules encompass structural genetic variations alongside dynamic expression patterns and sweeping regulatory reconfigurations [344].

To accelerate transfer, researchers must define modules through three core actions. First, must pinpoint the exact environmental threat, such as compounded heat and humidity or acidified soils. Second, must map the module to concrete quantifiable indicators like root architecture, heat recovery or disease resistance. Third, must isolate the minimum viable unit to aggressively target critical network bottlenecks and master regulatory switches rather than attempting exhaustive full‐pathway reconstructions. During cross‐species integration, adjusting regulatory components like promoters and tissue‐specific expression proves far superior to merely altering coding regions [345]. Utilizing CRISPR to edit these regulatory regions generates vital allele gradients that precisely calibrate traits and drastically minimize severe growth penalties, cementing regulatory modules as the ultimate entry point for transferring tropical resilience.

### 9.2 Donor Resource Exploration: From Tropical Crops to Wild Relatives

Targeting donor resources for cross‐species transfer must extend beyond cultivated varieties. Breeders actively prioritize hybridizable close relatives for directed introgression to rapidly enhance recipients possessing mature breeding systems. When cultivated populations lose critical traits, wild relatives provide a formidable alternative. Preserving the stress‐resistant backbone of wild species while rapidly restoring yield through multi‐locus editing empowers researchers to successfully engineer *de novo* domestication [346]. Additionally, local varieties from marginal ecological zones perfectly reflect real agricultural constraints and supply vital gene pools to achieve low‐cost adaptive trade‐offs.

Selecting these donor resources must actively advance a highly practical tranfer strategy rather than merely cataloging available genetic options. If donors and recipients easily intercross through a mature backcross system, directed introgression rapidly and cost‐effectively transfers key adaptive loci into high‐yield backgrounds. When hybridization fails or specific targets require reshaping homologous networks, researchers must shift toward functional replication by completely reconstructing homologous genes and complex regulatory frameworks within the recipient crop [347]. Furthermore, tropical resilience frequently relies on dynamic root and soil interactions alongside strategic management windows rather than plant genetics alone. Coupling synthetic microbial communities with precise agronomic management actively enhances system consistency and resilience across widely fluctuating tropical environments, establishing this approach as equally vital to direct genetic improvement. Consequently, cross‐species transfer strategies must firmly transition from purely tool‐oriented approaches to comprehensive decision‐oriented frameworks. This critical shift guarantees that researchers definitively select the most cost‐effective, highly verifiable and ultimately successful pathways for specific crop systems and complex target scenarios.

### 9.3 Four Engineering Strategies for Cross‐Species Transfer

For different crop systems, cross‐species transfer can be categorized into four strategies:

Strategy A: Genetic introgression. This is suitable for systems with cross‐compatible relationships with the donor, where traditional hybridization combined with molecular markers/GS can accelerate the transfer of key quantitative trait locus (QTLs)/genes [348]. The advantage of this strategy is that regulatory and acceptance aspects are relatively easier, but it is limited by linkage drag and breeding cycles.

Strategy B: Functional replication. When the recipient crop has homologous genes or networks, functional advantages from the donor can be replicated by editing the homologous loci of the recipient. This includes that Coding region editing: To obtain functional gain/loss or key amino acid substitutions. CRISPR editing of promoters/enhancers to generate a series of expression gradients or tissue‐specific allelic gradients that match optimal trade‐offs under compound stress, thereby reducing growth costs [349].

Strategy C: Regulatory rewriting and module stacking. Tropical adaptations often involve networks and single‐gene modifications are limited. Multiple genes need to be linked. Multi‐gene engineering and multi‐locus editing can achieve module stacking and pathway reconstruction, complemented by programmable regulation [347]. The rapid maturation of multi‐locus editing and multi‐gene engineering techniques provides a powerful tool for upgrading compound stress adaptation.

Strategy D: Microbiome and agronomy synergy. Under the high temperature and high humidity of tropical environments, soil‐borne diseases and root imbalances are more common, making pure genetic improvement difficult to cover all scenarios. By engineering the microbiome and precision agronomic management, field stability and system resilience can be enhanced [350]. This strategy is especially suitable for modules involving phosphorus acquisition, aluminum tolerance, disease resistance and heat and drought mitigation.

### 9.4 Closed‐Loop Assessment of Yield—Cost—Stability

There is a demand for the validation system to successfully transitioning cross‐species transfer from laboratories to tropical fields, which is securely anchored in yield stability and cost trade‐offs [351]. Relying entirely on enhanced stress tolerance under controlled conditions fails to guarantee stable agricultural gains. Researchers must aggressively shift validation from isolated assessments to comprehensive multi‐scenario evaluations that weigh yield, cost and stability equally. Yield metrics must expand beyond mere survival to explicitly target harvest and quality consistency. Cost assessments must capture obvious developmental delays alongside hidden penalties like heightened sensitivity to secondary threats. Furthermore, stability evaluations must confirm genetic repeatability across diverse backgrounds and ecological consistency in microbial colonization. Crucially, modular improvement requires iterative optimization. By deploying regulatory gradients and module stacking strategies, researchers can transform adaptive enhancements from rigid finalizations into adjustable continuous processes. This dynamic framework perfectly conquers the massive fluctuations and relentless compound stresses dominating tropical environments.

**FIGURE 7:**
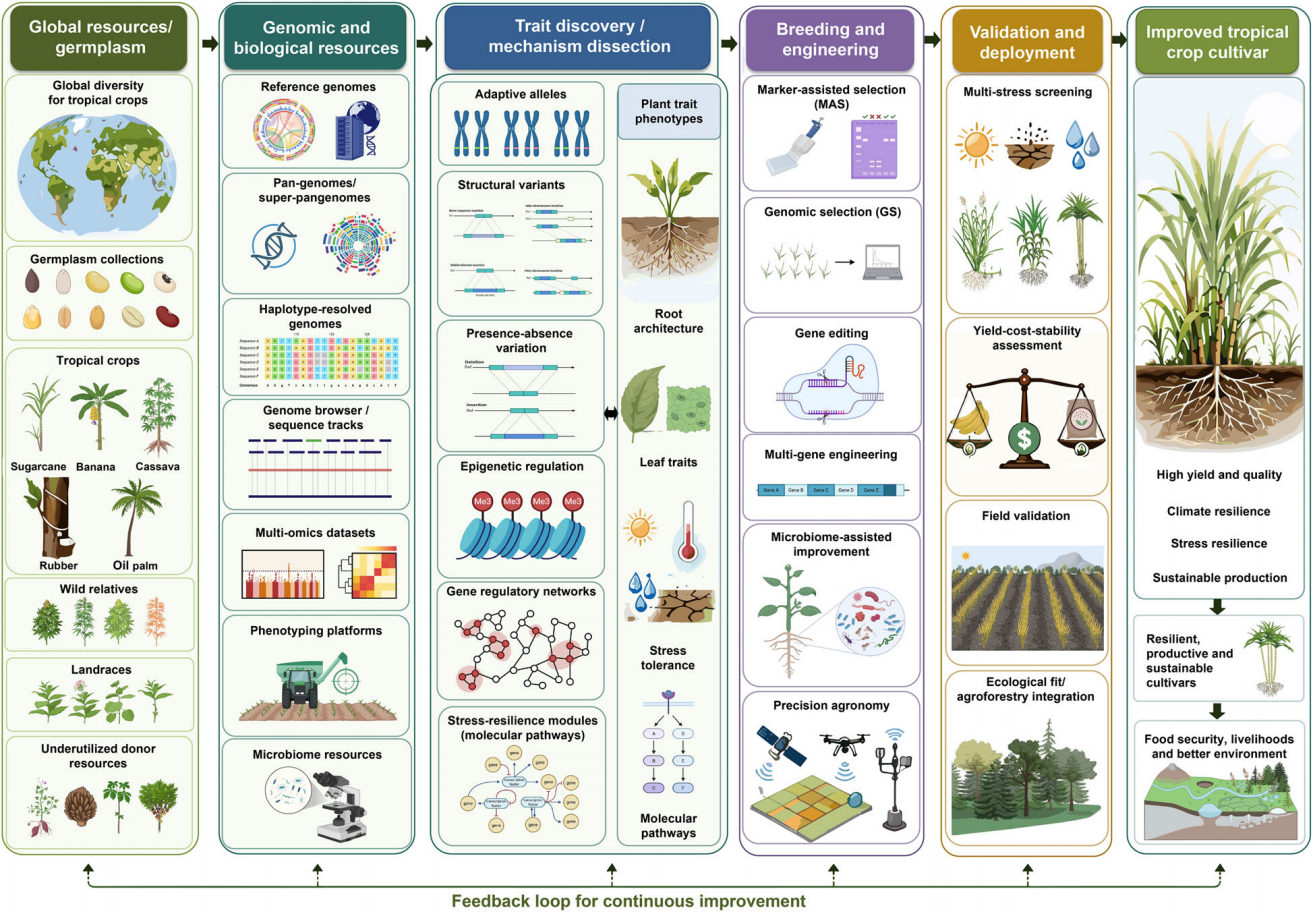
Integrated pipeline from global resources to the delivery of improved tropical crop cultivars. Schematic overview of a translational workflow linking resource discovery, genomic analysis, and breeding deployment in tropical crops. The pipeline begins with global germplasm resources, including tropical crop collections, wild relatives, landraces and underutilized donor resources. These are integrated with genomic and biological resources, such as reference genomes, pan‐genomes or super‐pangenomes, haplotype‐resolved genomes, multi‐omics datasets, phenotyping platforms and microbiome resources. The next stage focuses on trait discovery and mechanism dissection, including adaptive alleles, structural variants, epigenetic regulation and stress‐resilience networks. These discoveries are then translated into breeding and engineering applications, including marker‐assisted selection, genomic selection, gene editing, multi‐gene engineering, microbiome‐assisted improvement and precision agronomy. Candidate improvements are subsequently evaluated through multi‐stress screening, yield‐cost‐stability assessment, field validation and ecological fit. The pipeline ultimately leads to the delivery of improved tropical crop cultivars with enhanced climate resilience, productivity and sustainability. Figure created with BioRender.com.

Based on the above logic, we present a summary framework to facilitate transforming the tropical adaptive advantages summarized earlier into an executable roadmap (Table S2). The goal is not merely to reiterate common tools, such as genomics, gene editing or ecosystem design, but also to synthesize them into an integrated decision‐making structure for cross‐species transfer. Within this structure, each adaptive module is defined by a clear application scenario, measurable indicators, optional implementation pathways, associated costs and stability constraints. This framework transforms the evolutionary advantages of tropical crops into transferable and scalable resources for innovation. To further summarize this translational logic, we added an integrative workflow from global germplasm and genomic resources to breeding applications and the final delivery of improved tropical crop cultivars (Figure 7).

Tropical crops face unprecedented compound stresses from climate change. Their adaptive strategies, including polyploid genome plasticity, epigenetic memory, specialized root architectures and symbiotic microbial networks, provide a multi‑layered framework for climate‑resilient agriculture. Three priority directions are proposed for the next decade. First, establish super‑pangenome references for major tropical crops to catalog presence‑absence and structural variants from wild germplasm. Second, decode multi‑stress integration logic using time‑series multi‑omics and machine learning. Third, engineer minimal functional modules into target crops via gene editing and microbiome synergy, validated under realistic stress combinations. Achieving these goals requires trans‑disciplinary collaboration. The tropical crops community is well positioned to translate evolutionary insights into climate‑ready solutions for both tropical and temperate systems.

## Author Contributions

**WP, CHM, XJ and QY**: conceptualization, **WP and LC**: writing – original draft preparation, **WP, LC, WD, WQ, ZZ, GH, CH, CS, CHX, CHM, XJ and QY**: review and editing, **QY**: funding acquisition. All authors have read and agreed to the published version of the manuscript. All authors read and approved the final manuscript.

## Funding

This study was supported by the National Natural Science Foundation of China (XT, 82470065 and 82270077), R&D Program of Guangzhou National Laboratory (GZNL2025A02002), Project GZNL2025B01005 supported by Guangzhou National Laboratory and State Key Laboratory of Respiratory Disease, Innovative Drug Research and Development National Science and Technology Major Project (2026ZD1806500; 2026ZD1806501), Guangzhou Institute of Respiratory Health Open Project (XT).

## Conflicts of Interest

The authors declare no cnflicts of interests.

## Data Availability declaration

Data availability is not applicable to this article as no new data were created or analyzed in this study.

## Supporting information




**Supporting File 1**: advs76735‐sup‐0001‐SuppMat.docx.

**Supporting File 2**: advs76735‐sup‐0002‐TableS1‐S2.zip.
