# Supplementary material for: Adaptive Responses of Tropical Crops: A Multi‐Scale Omics Integrated Perspective
Source: Adv Sci (Weinh). 2026 Jul 27:e76735. Online ahead of print. doi: 10.1002/advs.76735 (PMC13403741; doi:10.1002/advs.76735)
Supplement: Supplementary file 1 — Supporting File 1: advs76735‐sup‐0001‐SuppMat.docx. [file ADVS-9999-e76735-s001.docx]

**Table S1. Public databases and AI-enabled digital resources supporting tropical crop research and breeding.**

| **Crop** | **Database** | **URL** | **Importance** | **Key Features** |
| --- | --- | --- | --- | --- |
| General | TropCRD | [www.tropical-resources.org.cn](http://www.tropical-resources.org.cn) | Comprehensive tropical crop data repository | Integrates germplasm, phenotype, trait info |
| General | TropiCODB | <https://github.com/daiwenba/TropiCODB> | Centralized tropical crop omics database | Genomic, transcriptomic, epigenomic data |
| General | TropGeneDB | <https://tropgenedb.cirad.fr/tropgene/JSP/index.jsp> | Crop genetics database | Molecular markers, QTLs, traits |
| General | TCOD | <https://ngdc.cncb.ac.cn/tcod/home> | Tropical Crop Omics Database | Multi-omics integration for breeding |
| Banana | MusaBase | <https://musabase.org/> | Banana genome and breeding data | Gene annotations, germplasm, traits |
| Cassava | Cassavabase | <https://www.cassavabase.org/> | Cassava genomics and breeding | SNPs, QTLs, phenotypes |
| Sugarcane | Sugarcane Genome Hub | <https://sugarcane-genome-hub.southgreen.fr/> | Sugarcane genomics and functional data | Genome assemblies, gene models, pangenome |
| Oil palm | PalmXplore | <https://ngdc.cncb.ac.cn/databasecommons/database> | Oil palm genome and trait database | Functional annotation, SNPs, candidate genes |
| Cocoa | CocoaGen DB | <https://agritrop.cirad.fr/> | Cocoa genome and trait database | Genomics, phenomics, trait association |
| Coffee | Coffee Genome Hub | <https://coffeegenome.org/> | Coffee genomics and breeding | Reference genomes, multi-omics |
| Rubber | HeveaDB | <http://hevea.catas.cn/home/index> | Rubber tree genomics and traits | Multi-omics, gene expression, markers |

**Table S2: Donor resource types, transferable traits, recommended improvement pathways, stress factors, and verification indicators.**

| **Donor resource type** | **Transferable traits/characteristics** | **Recommended improvement pathway** | **Main stress factors & combinations** | **Verification indicators (yield** & **cost** & **stability)** |
| --- | --- | --- | --- | --- |
| Wild relatives | Wild resistance & rapid cultivation | *De novo* domestication (multi-locus editing) | New crop base, extreme environments | Retained resistance; yield/agronomic trait completion |
| Salt-tolerant ecotypes, relatives | Salt tolerance/Ion stability (K^+^/Na^+^ balance) | Hybrid introgression or homologous recombination/editing | Saline land + high temperature/drought | Decreased leaf Na^+^, improved K^+^/Na^+^; increased yield |
| Flood-tolerant varieties/relatives | Flood/Anoxia tolerance (Recovery after flooding) | Introgression or homologous recombination | Flooding × high temperature | High survival rate; fast recovery; less yield reduction |
| Tropical crop advantage materials, local varieties | Low-cost "Heat/Wet resistance" regulation | Regulatory region editing (promoter/cis-elements) | High temperature × high humidity, heatwaves | More stable yield; no "slow growth/deformation" |
| Local varieties & advantageous rhizosphere microbes | Acidic soil, low P/Al resistance: Rhizosphere advantage | Microbial community & management | Low P × acidification/Al toxicity | Higher phosphorus uptake efficiency; stable microbial colonization; effective across multiple points |
| Disease-resistant resources, wild relatives | Disease pressure: Disease resistance without sacrificing growth | Regulatory optimization | High humidity, disease-prone areas | Reduced disease incidence; stable yield/quality; more durable resistance |

Note: The information summarized in this table is derived from a synthesis of the previously cited literature and the authors' domain expertise. The donor resource types, transferable traits, improvement pathways, stress factors, and verification indicators are intended as a conceptual framework for cross-species translation and breeding design, rather than as point-by-point conclusions from a single source.
